# TMEM203 Is a Novel Regulator of Intracellular Calcium Homeostasis and Is Required for Spermatogenesis

**DOI:** 10.1371/journal.pone.0127480

**Published:** 2015-05-21

**Authors:** Prashant B. Shambharkar, Mark Bittinger, Brian Latario, ZhaoHui Xiong, Somnath Bandyopadhyay, Vanessa Davis, Victor Lin, Yi Yang, Reginald Valdez, Mark A. Labow

**Affiliations:** Novartis Institutes for Biomedical Research, Developmental and Molecular Pathways, 100 Technology Square, Cambridge, Massachusetts, United States of America; University of Nevada School of Medicine, UNITED STATES

## Abstract

Intracellular calcium signaling is critical for initiating and sustaining diverse cellular functions including transcription, synaptic signaling, muscle contraction, apoptosis and fertilization. Trans-membrane 203 (TMEM203) was identified here in cDNA overexpression screens for proteins capable of modulating intracellular calcium levels using activation of a calcium/calcineurin regulated transcription factor as an indicator. Overexpression of TMEM203 resulted in a reduction of Endoplasmic Reticulum (ER) calcium stores and elevation in basal cytoplasmic calcium levels. TMEM203 protein was localized to the ER and found associated with a number of ER proteins which regulate ER calcium entry and efflux. Mouse Embryonic Fibroblasts (MEFs) derived from Tmem203 deficient mice had reduced ER calcium stores and altered calcium homeostasis. Tmem203 deficient mice were viable though male knockout mice were infertile and exhibited a severe block in spermiogenesis and spermiation. Expression profiling studies showed significant alternations in expression of calcium channels and pumps in testes and concurrently Tmem203 deficient spermatocytes demonstrated significantly altered calcium handling. Thus Tmem203 is an evolutionarily conserved regulator of cellular calcium homeostasis, is required for spermatogenesis and provides a causal link between intracellular calcium regulation and spermiogenesis.

## Introduction

Calcium is a ubiquitous second messenger that controls a large number of functions, both cell specific, such as muscle contraction and synaptic activity, and broad, such as modulation of gene transcription and apoptosis [1]. Central to its use as a second messenger, cytoplasmic calcium concentrations are kept very low (less than 0.1μM) by actively pumping calcium out of the cell and into intra-cellular stores, the best characterized of which is the endo(sarco)plasmic reticulum (ER) where calcium concentration are ~ 1 mM [2]. The ER calcium reserve is largely achieved by the ATP dependent ER calcium pump, SERCA (sarcoplasmic/endoplasmic reticulum calcium ATPase), that continuously pumps calcium into the ER. The ER provides a store that allows rapid calcium release to trigger a large array of responses to extracellular signals. In addition, the Plasma Membrane Ca^2+^ ATPase (PMCA) family of membrane pumps are involved in extruding cytosolic calcium and thus helps in achieving low cytosolic calcium concentrations [3].

Two of the general functions of calcium are to regulate rapid transcriptional responses and cell death and apoptosis. Calcium dependent transcription is critical for control of a variety of immunologic, neurologic and metabolic functions through the Nuclear Factor of Activated T cells (NFAT) and cAMP Responsive Element Binding (CREB) regulated transcription co-activator/ Transducer of regulated cAMP response element-binding protein (CRTC/TORC) transcription factors [3–9]. Upon stimulation of a variety of receptors including, the B- and T-cell antigen receptors, tyrosine kinase receptors and G Protein Coupled Receptors (GPCRs), phospho-lipase C (PLC) is activated which, in turn, leads to production of inositol 1, 4, 5-triphosphate (IP3) and diacylglycerol (DAG). IP3 binds and activates the IP3 Receptor (IP3R), an ER localized calcium conducting channel. The resulting depletion of the ER calcium store is sensed by a single pass EF domain containing calcium sensor protein called stromal interaction molecule 1 (STIM1), which then oligomerizes and interacts with the plasma membrane localized Calcium Release Activated Calcium (CRAC) channel, CRAC Modulator (known as ORAI or CRACM) on the plasma membrane. Orai proteins are four-pass plasma calcium channels that, upon oligomerization, allow rapid entry of extracellular calcium. This influx of calcium triggered by ER depletion is termed store operated calcium entry (SOCE) [2,10–13]. This leads to a more sustained increase in cytoplasmic calcium which activates the calcium dependent phosphatase, calcineurin which dephosphorylates NFAT and CRTCs allowing their nuclear re-localization. ER calcium stores are also key regulators of cellular apoptosis. Low levels of calcium release by the ER, through a B-cell lymphoma protein 2 alpha (BCL-2) dependent mechanism are thought to promote cell survival, while large releases of ER-calcium likely result in apoptosis in a BCL2 associated X protein (BAX)/ BCL2 antagonist killer (BAK)dependent manner[14,15]. The complexity of regulation of calcium stores and its link to apoptosis is illustrated by the observation that individual members of the BCL-2 family appear to act both to increase and decrease ER-calcium stores depending on the physiologic setting [14,15].

Intracellular calcium plays critical roles in fertility as well [16]. Many recent studies have shown that intracellular calcium is critical for sperm motility, capacitation, and the acrosome reaction [17–19]. Fusion of spermatozoa with an oocyte is followed by activation of the fertilization process that begins with Ca^2+^ oscillations in the egg [16,20]. Intracellular calcium is also involved in the progression of meiosis in mammalian oocytes [21]. The role of intracellular calcium in the regulation of the spermatogenesis, however, is unknown. Various calcium mobilizing channels/pumps, calmodulin and other calcium binding proteins are differentially expressed during mammalian spermatogenesis and in the support cells suggesting that calcium could be involved in the regulation of mammalian spermatogenesis [18,22].

Spermatogenesis is the process of formation of mature spermatozoa from primordial germ cells in the testes. It is a complex process that can be broadly divided into three stages involving mitosis, meiosis and spermiogenesis. In the first stage, spermatogonia, germ-cell precursors undergo mitotic divisions to produce primary spermatocytes. These are diploid cells that are committed to meiosis and give rise to four haploid round spermatids. In spermiogenesis, the round spermatids differentiate into species-specific shaped spermatozoa, with dramatic morphological changes, including elongation and condensation of the nucleus, and formation of the flagellum. Finally, the mature sperm is released into the tubules by a process called spermiation. At all stages of differentiation, the spermatogenic cells are in close contact with Sertoli cells which provide structural and metabolic support to the developing sperm cells. Spermatogenesis is regulated by a variety of hormones and local factors. The molecular mechanisms involved in the regulation of these different stages of mammalian spermatogenesis have been aided by the study of a large number of mouse knockout strains though the roles of second messengers, and calcium in particular are not well understood [23–27].

We have used a cDNA over-expression strategy to discover proteins capable of regulating intracellular calcium signaling. This report describes a previously uncharacterized predicted multi-pass trans-membrane protein (TMEM203) whose exogenous expression induced nuclear localization of both CRTC1 and NFAT due to increased cytoplasmic calcium levels and activation of calcineurin. TMEM203 is a highly conserved ER protein which associates with SOCE components. Inhibition of Tmem203 expression reduced basal ER calcium stores. Tmem203 deficient male mice were sterile and exhibited a profound defect in spermatogenesis and spermiogenesis. Concomitant with the loss of spermiogenesis, loss of Tmem203 resulted in a profound deficit in the ability of spermatocytes to maintain cytoplasmic calcium concentrations after stimulation. Thus, TMEM203 is a novel regulator of intracellular calcium homeostasis and is required for spermatogenesis.

## Materials and Methods

### Constructs and reagents

TMEM203; D1ER, NFAT2 (1–402) were synthesized and cloned into desired plasmids by Genscript USA Inc. pTUNE-TMEM203, RFP-fused organelle markers were purchased from OriGene Technologies Inc. Various antibodies were purchased from respective companies as mentioned—STIM1 (Cat # 4916) and SERCA2 (Cat # 4388) from Cell signaling technologies. IP3R antibody (# 610312) from BD Bioscience, INSIG1 (Cat #ab70781) from Abcam and M2 FLAG from Sigma Aldrich. siRNA against Human TMEM203 were from Qiagen—siRNA TMEM203—CAGGCACTGCTTGGCTTACTA (Cat #-SI00633185); control siRNA were purchased from Dharmacon-Thermo Scientific—Non-Targeting siRNA #1(Cat #-D-001210-01-05).

### Animal Use

All animal procedures were approved by the Institutional Animal Care and Use Committee of Novartis Institute for Biomedical Research and were in compliance with the US regulations (Guide for the Care and Use of Laboratory Animals, Eight Edition 2011).

### NFAT Luciferase assay

HEK293 cells were transfected with indicated plasmids in combination with 10 ng TK Renilla, test and/or empty vector, and 50 ng NFAT-Luc (Stratagene Inc). Transfections were carried out in 96 well format using approximately 25,000 cells per well. Cells were exposed to either DMSO, 5 nM CsA, 1 μM PMA for 8 hours. Reporter activities were determined 48 hours after transfection with the Dual-Glo luciferase reagent as per manufacturer's instructions (Promega).

### Western blotting

Lysates for analysis from various cells were prepared with 1X Cell Lysis Buffer (Cell Signaling Technology Cat #9803) containing 20 mM Tris-HCl (pH 7.5),150 mM NaCl, 1 mM Na_2_EDTA, 1 mM EGTA, 1% Triton, 2.5 mM sodium pyrophosphate, 1 mM beta-glycerophosphate, 1 mM Na_3_VO_4_ and1 μg/ml leupeptin with addition of 1 mM PMSF. The lysed cells were collected and centrifuged at 4^°^C at 13K for 10 mins. The lysate was transferred to a new tube and mixed with NuPAGE loading buffer (NP0007—Invitrogen). Lysates were heated for 10 mins at 70^°^C and subjected to gel electrophoresis on a NuPAGE Novex 4–12% Bis-Tris Gel (Invitrogen) by following manufacturer’s instructions and transferred to a nitrocellulose membrane using NuPAGE transfer system (Invitrogen). After transfer, the membrane was blocked with blocking buffer (Licor Bioscience), washed and blotted with indicated antibodies at 4^°^C for 24 hours. Next, the blot was incubated with appropriate secondary HRP/IR-dye-conjugated antibodies were incubated for 45 minutes. After further washing with TBST, the membrane was developed using ECL reagents or IR reader (Licor Bioscience).

### Immunoprecipitation

1 million HEK293 cells were seeded onto 6 well plate. Next day, the cells were transfected with pFLAG-CMV-TMEM203 or empty vector (4μg/well using fugene HD (Roche)). After allowing 24 hrs for expression the cells were lysed with 1X Cell Lysis Buffer (Cell Signaling Technology Cat #9803). For TMEM203 or STIM1 interacting protein co-immunoprecipitation, lysates obtained after centrifugation at 4^°^C were incubated with EZview Red ANTI-FLAG M2 (Sigma-Aldrich Cat # F2426) or anti-STIM1 antibody (Cell signaling technologies) or Rabbit IgG bound to Protein G beads (Invitrogen) for immune-precipitation for overnight (~16 hrs) with constant mixing. Beads were then washed three times with 1X cell lysis buffer. The proteins were eluted with 2X sample SDS-PAGE loading buffer. IP elute were boiled for 10 minutes at 70^°^C and analysed using western blotting.

### Ca^2+^ Flux Measurements

Cytosolic Ca^2+^ Flux was measured using flow cytometry. Breifly, cells were loaded for 30 min at 37^°^C with 2μM indo-1 acetoxylmethylester (Indo-1 AM) (Molecular Probes) in Hanks’ balanced salt solution (HBSS) (Invitrogen Cat # 14025) with 1% FCS and 2.5mM probecinide (Invitrogen). Cells were washed once and resuspended in calcium and magnesium free HBSS (Invitrogen # 14175) with 1% FCS. To measure intracellular store calcium flux cells were treated with 1mM EGTA and subsequently with thapsigargin, ionomycin, or m-3m3FBS (Tocris Bioscience) and changes in fluorescence intensity were monitored on an LSRII flow cytometer (BD Biosciences). Values were plotted as the ratio of fluorescence at Ca^2+^-bound Indo-1 AM to that of Ca^2+^-free Indo-1 AM. Data were analyzed with FlowJo software (TreeStar).

### [Ca^2+^]_i_ measurements

cDNA transfected HeLa Cells were seeded onto coverslips and incubated overnight. Cells were loaded with 4 μM Fura-2 acetoxymethyl (AM) ester (Molecular Probes) at room temperature for 30 mins in HBSS (Invitrogen Cat # 14025) with 20mM Hepes. After one wash the cells were further incubated in HBSS with 20mM Hepes to allow de-estrification for 20 mins. The coverslips were mounted onto chamber and measuremnts were performed using a Xenon light source filtered at 340 nm and 380; emission (λ > 480 nm) was captured by a cooled CCD camera (Hamamatsu Photonics; SlideBook software, Olympus). Background-corrected 340/380 ratio images were collected and calcium concentration were calculated.

### Single Cell D1ER FRET

D1ER stable HEK293 cells transfected with siRNA (~4–5 days) were imaged using an inverted Olympus IX81 fluorescence microscope. The cells were imaged in HBSS (Invitrogen # 14025), with 20 mM HEPES (pH 7.4), 1mM CaCl_2_ or calcium and magnesium free HBSS (Invitrogen Cat # 14175) with 20 mM Hepes, 0.5mM EGTA and 1mM MgCl_2_ at room temperature. Cells were alternatively excited with 340 and 380 nm light (Lambda DG-4; Sutter Instrument Co.) for 100–200 ms, and the emission >510 nm was captured using a CCD camera (Orca-ER; Hamamatsu Photonics) and analyzed with Slidebook (Intelligent Imaging Innovations). After background subtraction, the ratio of the 340- and 380-nm excited images were collected.

### Generation of *Tmem203* null mice

*Tmem203* resides immediately upstream of exon 1 of Ndor1 (NADPH dependent diflavin oxido-reductase 1) in anti-sense orientation. It is also 6 kb upstream of a putative gene C430004E15Rik in the sense orientation. Therefore the targeting construct was designed to maximize disruption of the *Tmem203* allele while preserving the functionalities of the two neighboring genes. In the KO+neo allele, the translational initiation ATG of *Tmem203* as well as 44 nt of its 5’ UTR was replaced with a FRT flanked neo cassette. After removing the neo cassette, a fragment of 102 nt from the vector including one FRT site remains in the genome (KO). The targeting construct had a 4.7 kb 5’ homology arm and a 2.7 kb 3’ homology arm surrounding a FRT flanked neo cassette. The targeting construct was transfected into mouse C57BL/6 embryonic stem cells and G418 resistant colonies were selected and homologous recombination events were identified by standard genomic PCR and Southern-blot methods. Correctly targeted ES clones were injected into C57BL/6J host blastocysts and germline transmission was followed. Subsequently, heterozygous mice were crossed with Flpe deleter mice to remove the neo cassette.

Genomic PCR was used to amplify relevant regions and determine the genotypes of mice. The genotyping primers were 5’- agcaggcggtcgccaggtctgc (forward) and 5’ caccaacagggccagcaggtgc (reverse). Further, Tmem203 gene disruption was confirmed directly by sequencing the cDNA from *Tmem203* null mice lung tissue. Primers used to amplify Tmem203 cDNA were 5 ‘gcttctgctagcctccggttccg (forward) and 5’ ggagtcaagccatccactcgc (reverse)—Normal expression of Ndor1 was detected by western blotting in Tmem203 lung tissue lysates (data not shown).

### Uninterrupted Mouse mating

To assess fertility, Tmem203 null and heterozygote male and female mice at 8 weeks of age were mated with Wild type mice at 1:1 ratio for a period of 19 weeks. The number and size of litters of offspring were recorded for each breeding pair before weaning.

### Analysis of spermatozoa

Epididymides were removed from Tmem203 null and wild type male mice at 20 weeks of age. The cauda region from epididymis was cut open and incubated in a defined volume of PBS with 10% fetal calf serum at 37°C for 10 min. A homogeneous suspension was then diluted and analyzed using HTM-IVOS motility analyzer (version 10.8; Hamilton Thorne Biosciences, Beverly, MA) for computer assisted sperm analysis (CASA) and by a hemacytometer. For CASA, data was collected with the following default settings: phase contrast; frame rate, 60 Hz; minimum contrast, 30; low and high static size gates, 0.8 and 6.25; low and high intensity gates, 0.25 and 1.50; low and high static elongation gates, 20 and 70; default cell size, 5 pixels; default cell intensity, 55; magnification, ×0.78. For each genotype, sperm from two epididymides were examined, and data were averaged from 20 fields with a total of >500 sperm. The sperm motility was classified using average path velocity (VAP) parameter as—fast (VAP > 100 μm/s); Medium (VAP = 100–50 μm/s); slow (VAP = 50–10 μm/s) and immotile (VAP < 10 μm/s).

### Gross Pathology and Histopathology

For histopathology studies tissues were harvested at necropsy from 38-week-old or 48-week-old male mice (testes, epididymides and prostate gland). Tissues harvested at necropsy were fixed in modified Davidson’s fixative [28] for approximately 24 hours then routinely processed, embedded in paraffin, sectioned at 5.0 μm, stained with hematoxylin and eosin (H&E) and evaluated by bright field light microscopy. A Periodic acid-Schiff special tissue staining kit (Sigma; # 395B) was used to stain testes tissues in order to facilitate staging of seminiferous tubular epithelium which was accomplished using well established criteria described previously [29–31]

### Transmission Electron Microscopy

For ultrastructural studies testes were harvested from 32-week-old male mice and fixed overnight at 4°C in a solution consisting of 2% gluteraldehyde, 2.5% paraformaldehyde and 0.06% picric acid in 0.2M sodium cacodylate buffer (pH7.4). Fixed specimens were washed with cacodylate buffer, post-fixed in osmium tetroxide, dehydrated in graded ethanol and embedded in epoxy resin. Semithin sections were cut at approximately 0.35 μm and stained with toluidine blue and basic fuchsin and examined by light microscopy for regions of interest. Ultrathin sections (90 nm) were stained with uranyl acetate and lead citrate and examined with a Tecnai G^2^ Spirit BioTWIN transmission electron scope equipped with an AMT 2k CCD camera.

### Microarray Studies

For microarray studies left and right testes were individually harvested from control and *Tmem203* null mice at 24 weeks of age. At necropsy the left and right testis were individually weighed and recorded along with individual body weights and brain weights in order to assess the combined left and right testes weights relative to body and brain weights. Total RNA was extracted from the testes using RNAasy miniprep kit (Qiagen). Transcriptional profile of each of the nine samples (4 wild type testes and 5 null mice testes) was probed by using Affymetrix Mouse Genome 430 2.0 GeneChips. The raw data obtained after scanning the arrays were assessed for quality by array QualityMetrics [32], a Bioconductor (http://bioconductor.org/) package for quality assessment of microarrays. Robust Multichip Average (RMA) normalization consisting of three steps: a background adjustment, quantile normalization and finally summarization, was applied before obtaining model-based gene expression indices, also known as signal values [33]. Principal Component Analysis was applied on the RMA normalized data to assess for intra group versus inter group variability. All samples were retained for further downstream analyses. All profiling data has been deposited in the Geo Database (http://www.ncbi.nlm.nih.gov/geo/) with the geo accession number GSE66791.

To identify differentially expressed genes between wild type and null testes samples, LIMMA (Linear Models for Microarray Analysis), a Bioconductor (http://bioconductor.org/) package for assessing differentially expressed genes from microarrays using linear models and empiral Bayes methods, was used [34]. All the analyses were done at probeset level. A probeset was retained for further analyses if it showed a 1.5 fold change in the wild type vs null comparison at p < 0.05 (after adjustments for false discovery rate). Additional filters for detection (signal ≥ 32 in at least one chip for a given probeset) and probeset quality (probesets mapping uniquely to only one gene) were applied to account for high quality probesets.

To identify differentially regulated pathways in the null vs wild type testes, pathways analysis (at gene level) was performed. Only one probe set per gene was selected based on the most significant differential p-value. To this end, pathway was performed using IPA (Ingenuity Systems, www.ingenuity.com).

### Testicular cell analysis by PI staining

Testes were dissected and decapsulated to release the tubules in HBSS (Invitrogen # 14025). The tubules were allowed to settle and excess HBSS removed. The tubules were incubated in 0.25 mg/ml (0.25%) collagenase (Worthington) at 32^°^C for up to 30 min with agitation. Dispersed tubules were allowed to settle and washed twice to remove peritubular cells. Washed tubules were then incubated with 0.25 mg/ml trypsin (GibcoBRL) and 1 mg/ml DNaseI (Roche) at 32^°^C for 10 min with agitation. Trypsin digestion was terminated by adding an equal volume of HBSS with 10% FCS. The suspension was centrifuged at 400 g for 5 min and tubules resuspended in HBSS with 1% FCS and disaggregated into a single-cell suspension by repeated pipetteting. Aggregates were removed by filtering the cell suspension through a 50 mm Nitex filter. Cells were then re-suspended in a defined volume of HBSS 1% FCS and counted. 0.2 million testicular cells were rinsed re-suspended in 0.2 ml of cold PI staining solution (10mM Tris, pH 8.0, 1mM NaCl, 0.1% Nonidet P40, 50 mg/ml PI, 10 mg/ml RNaseA), vortexed for 2–3 s and incubated on ice for 10 min to lyse the plasma membrane and stain nuclear DNA. Nuclear size and complexity were determined by FACScan analyzer using LSRII (BD Biosciences).

### RNA Isolation, Real-Time Reverse Transcriptase Polymerase Chain Reaction and Analysis of Transcript Levels

Same RNA preparations were used for microarray study and data confirmation. 2ug of RNA was reverse transcribed using High Capacity cDNA RT kit (Applied Biosystems). For expression analysis in Mouse tissue’s cDNA were purchased from Clonetech (Mouse MTC panel). Real time PCR reactions were performed using TaqMan Universal PCR Master mix in an ABI7500 Fast Real-Time PCR system as per manufacturer's instructions (Applied Biosystems). Average threshold values (CT) from three to four PCRs were determined for target genes, and these values were normalized to average GAPDH (ΔCT). Changes in gene expression were calculated as-fold change compared with control samples using the comparative ΔΔCT method. Taqman gene expression assays (all from Applied Biosystems): transmembrane protein 203 (*Tmem203*)-[Mm_00616517_s1]; inositol 1,4,5-triphosphate receptor 1 (Itpr1)-[Mm00439907_m1]; ATPase, Ca++ transporting, plasma membrane 1 (Atp2b1)-[Mm01245805_m1]; transient receptor potential cation channel, subfamily V (Trpv6)-[Mm00499069_m1]; transient receptor potential cation channel, subfamily M, member 5 (Trpm5)- [Mm01129032_m1]; transient receptor potential cation channel, subfamily M, member 8 (Trpm8)—[Mm01299593_m1]; calcium channel, voltage-dependent, gamma subunit 5 (Cacng5)-[Mm00519145_m1]; with controls glyceraldehyde-3-phosphate dehydrogenase (GAPDH) [4352339E]. Transmembrane protein 203 (TMEM203)–[Hs_00540709_s1]. For TG and Iono treated MEF cells the following taqman gene expression assays were used—Careticulin (Calr) [Mm_00482936_m1], Calcitonin receptor (Calcrl) [Mm_00516989_m1].

### Calcium flux with testicular cells

Adapted and modified from reference-[35]. 5–10 million testicular cells (obtained as described above) were stained with 5μM Fluo3-AM (Molecular probes Cat # F14218) and 10μM Fura-red-AM (Molecular probes Cat # F3021) in the presence of 4 mM probenecid (Molecular probes Cat # P36400) for 30 mins at room temperature in HBSS containing calcium and magnesium (Invitrogen Cat # 14025) with 1% FCS. The cells were then washed and re-suspended in HBSS without calcium and magnesium (Invitrogen Cat # 14175) with 1% FCS and incubated for 20 mins. Intracellular calcium measurements were performed in the presence of 1 mM EGTA in response to Thapsigargin, Ionomycin and m-3MFBS (Tocris Bioscience). Ratiometric calcium flux measurements were performed by FACScan using LSRII (BD Biosciences) by collecting Fluo3 calcium bound versus Fura-red calcium free ratio. The data was analyzed using FloJo and equivalent populations consisting of predominately round spermatids were gated for analysis [36,37].

### Statistical analysis

Unpaired two-tailed Student’s t-tests yielded P-values as listed. P values were considered significant at p < 0.01.

## Results

### TMEM203 expression activates calcineurin dependent transcription factors by elevating basal calcium levels

**Fig 1:**
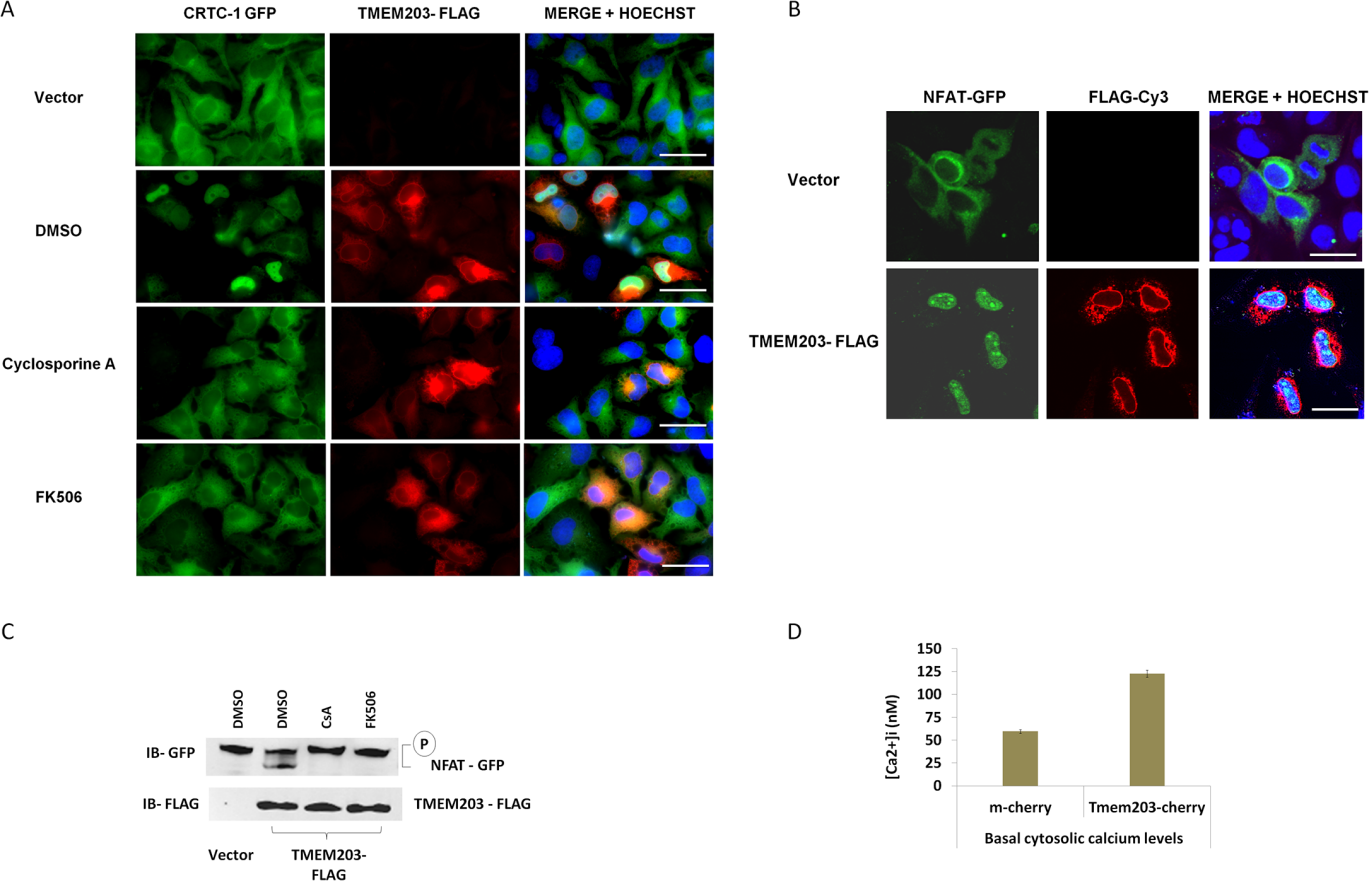
TMEM203 expression drives calcineurin dependent transcription factor activation by elevating the basal cytosolic calcium levels in HeLa cells. (A) Stably expressed CRTC1-GFP localization was visualized using fluorescent microscope in HeLa-CRTC1-GFP cell line transiently expressing TMEM203–FLAG for 48 hrs. CRTC1-GFP (green) nuclear translocation was induced in cells co-expressing TMEM203-Flag (red). Nuclei (blue) were visualized with Hoechst. Nuclear translocation was inhibited by treatment with 5nM Cyclosporine A or 10nM FK506 for 2 hour prior to fixing the cells. Scale bars = 15 μm. (B) HeLa cells were co-transfected with NFAT2 (1–402)-GFP and TMEM203-FLAG or empty vector. 48 hours later the cells were visualized using fluorescent microscope. Scale bars = 15 μm. (C) HeLa cells were co-transfected with NFAT2 (1–402)-GFP and TMEM203-FLAG or empty vector as indicated. 48 hours later the cells were treated with 5nM Cyclosporine A (CsA) or 10nM FK506 for 2 hours and total cell lysates were prepared. The lysates were subjected to immunoblotting with indicated antibodies. (D) TMEM203-mcherry or mcherry transfected HeLa cells were seeded onto coverslips and single cell Fura-2 fluorescence based calcium measurements were performed. The measurements showed elevated basal calcium levels in TMEM203-mcherry expressing cells. (Mean; +/- SE; n = 64 cells (mcherry); 55 cells (TMEM203-mcherry) from multiple coverslips; p value = 4.06719E-30).

Previously we described a high content microscopy based cDNA screen to identify genes that induced nuclear translocation of the CREB coactivator, CRTC1 [8]. This and other work demonstrated translocation of the CRTC1, is induced by elevations in cAMP or intracellular calcium through rapid calcineurin dependent dephosphorylation of CRTC1 [6,8]. In addition to known regulators of calcium and cAMP signaling, several novel proteins were identified and retested here for the ability to induce nuclear translocation of CRTC1. Of the genes analyzed, TMEM203 transfection resulted in efficient CRTC1 translocation without inducing gross morphologic and/or apoptotic changes (S1 Table). As shown in Fig 1A, exogenous expression of TMEM203 in CRTC1-eGFP expressing HeLa cells resulted in nuclear translocation which was blocked by the calcineurin inhibitors Cyclosporine A (CsA) and FK506. Addition of the calcium chelator, EGTA, also blocked TMEM203 induced nuclear CRTC1, and this inhibition was reversed by the addition of excess calcium (data not shown). In addition expression of TMEM203-FLAG induced nuclear localization of NFAT1(1–402)-GFP in cotransfected HeLa cells (Fig 1B). TMEM203 expression resulted in production of de-phosphorylated NFAT-GFP which was blocked by CsA or FK506 (Fig 1C).

Consistent with activation of calcineurin, TMEM203 over-expression increased intracellular calcium. After transfection of an m-cherry-TMEM203 construct, HeLa cells maintained significantly higher basal cytosolic calcium levels as compared to m-cherry transfected cells (Fig 1D). TMEM203 expression induced NFAT dependent transcription as indicated by an NFAT dependent reporter gene assay in the presence or absence of PMA (S1 Fig). Reporter induction was inhibited by CsA as well as by SKF96365, a potent inhibitor of extracellular calcium entry thought to inhibit ORAI1. These observations strongly suggest that TMEM203 expression leads to SOCE, increased cytoplasmic calcium concentrations, calcineurin activation and subsequent dephosphorylation and nuclear translocation of CRCT1 and NFAT.

### TMEM203 encodes a conserved ER resident protein associated with calcium signaling molecules

*TMEM203* cDNAs and gene locus encode a potential 136 amino acid integral membrane protein with four predicted trans-membrane (TM) domains, short N- and C-terminal domains with no obvious functional domains (S2 Fig). The predicted TMEM203 protein is remarkably conserved across vertebrate species while predicted proteins of much lower similarity were identified from *Drosophila* and *C*. *elegans* (39% and 27% identity, respectively; data not shown).

**Fig 2:**
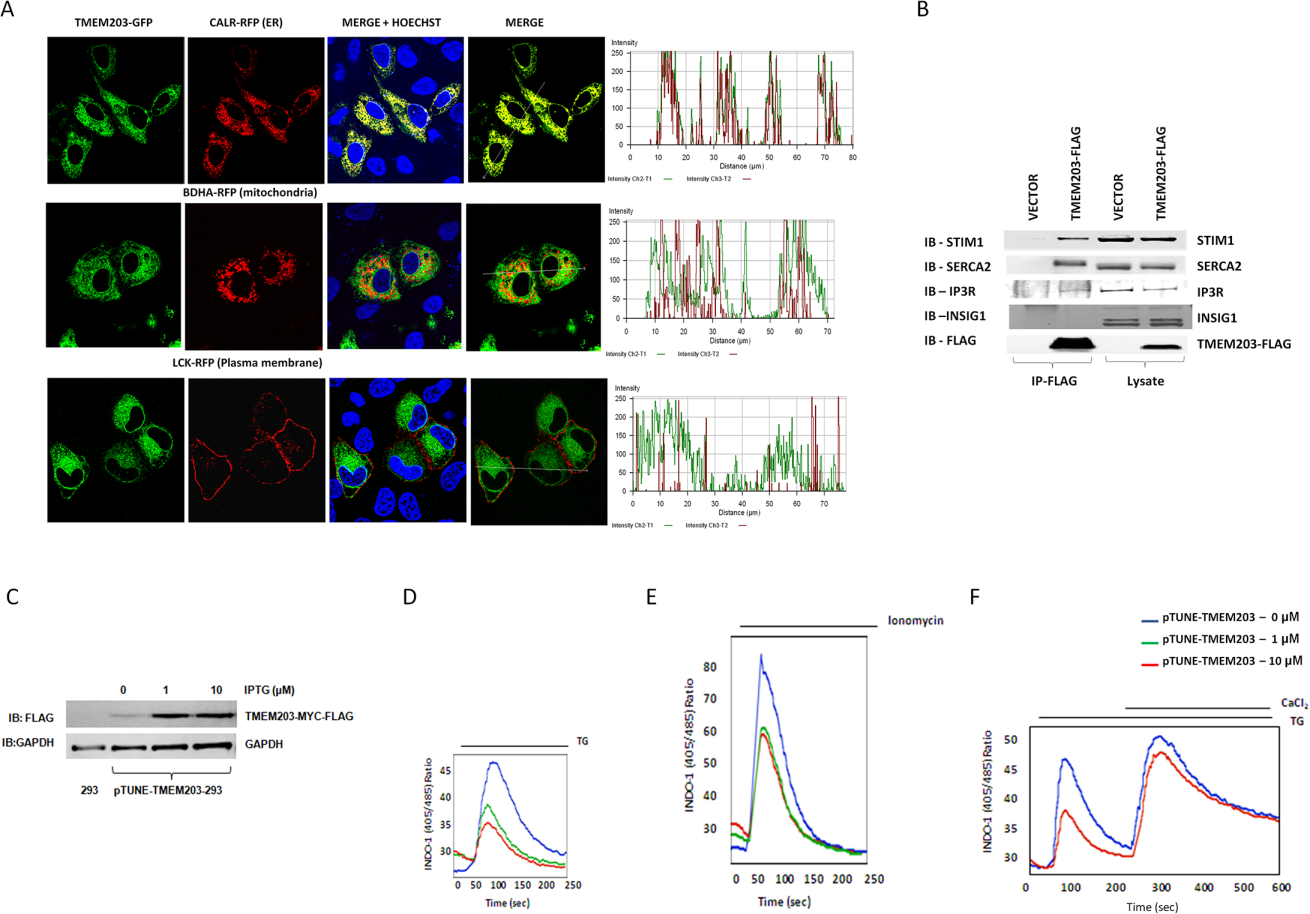
TMEM203 interacts with regulators of ER calcium stores and overexpression depletes ER calcium stores. (A) Confocal analysis of HeLa cells transiently expressing TMEM203-GFP with organelle specific markers for ER (top:Calreticulin-RFP), Mitochondria (middle:BDHA-RFP) or plasma membrane (bottom:LCK-RFP). Separation or colocalization of TMEM203-GFP and organelle marker(s) were visualized by the linescan function of MetaMorph: the fluorescence intensity of each pixel of the line of interest (white lines ~ 75 μm) is shown as a xy-graph for the corresponding green and red channels. The line scan shows that TMEM203-GFP predominately overlapped with the ER marker. (Representative of ~ 50 cells from 2 independent experiments). Note, we cannot rule out that TMEM203 is completely absent from the the mitochondria. (B) Western analysis of complexes immune-precipitated TMEM203-Flag from HEK293 cells with indicated antibodies shows specific interaction with endogenous STIM1, IP3R and SERCA2. (Representative of atleast 2 independent experiments). (C) pTUNE-TMEM203-293cells were treated with the indicated dose of IPTG for 48 hrs to induce TMEM203 expression. Levels of TMEM203-Flag protein were detected by western blot. (D) These IPTG induced cells were subjected to Indo-1 based calcium flux measurements by flow cytometry by first treating with thapsigargin (TG) and EGTA. (E) As in (D) but the cells were treated with Ionomycin. (F) As in (D) but following TG treatment CaCl_2_ was added to record SOCE.

Cellular localization of a TMEM203-GFP fusion protein was examined by imaging with ER, mitochondria and plasma membrane (PM) markers. Based on the linescan co-lozalization tool TMEM203-GFP was predominately co-localized with the ER marker protein in HeLa cells (Fig 2A). Considering the localization of TMEM203 and TMEM203’s effect on cytoplasmic calcium levels, we asked if TMEM203 was associated with calcium modulatory proteins complexed within the ER [38–40]. Endogenous IP3R, SERCA2 and STIM1 proteins were co-precipitated with TMEM203-FLAG expressed in transfected HEK293 cells, whereas an ER resident membrane protein unrelated to the SOCE complex, INSIG-1 was not (Fig 2B). TMEM-203-FLAG protein was also detected after immunoprecipitation with antibody to endogenous STIM1 (S3 Fig). Thus ER expressed TMEM203 was associated with proteins critical for regulation of calcium influx and efflux into the ER as well STIM1, the calcium sensor for SOCE.

### Overexpressed TMEM203 depletes ER calcium stores

TMEM203 might increase cytoplasmic calcium levels via either directly activating SOCE or via lowering ER calcium stores. To distinguish between these models, we first examined ER calcium stores indirectly by measuring the increase in cytoplasmic calcium levels after release of ER stores. HEK293 cells stably containing an IPTG-inducible TMEM203 construct were treated with various levels of IPTG and the ER flux into the cytosol was measured in the absence of extracellular calcium after exposure to thapsigargin (TG) or ionomycin. IPTG induced TMEM203 expression (Fig 2C) resulted in significantly lowered calcium release from the ER in response to both TG and ionomycin (Fig 2D–2F). This demonstrates that TMEM203 overexpression causes a significant depletion of ER calcium stores. Influx of extracellular calcium after TG induced store depletion was unaffected by TMEM203 over-expression (Fig 2F) suggesting that TMEM203 does not affect SOCE directly.

### TMEM203 is required for maintaining normal ER calcium level and calcium dependent gene expression

**Fig 3:**
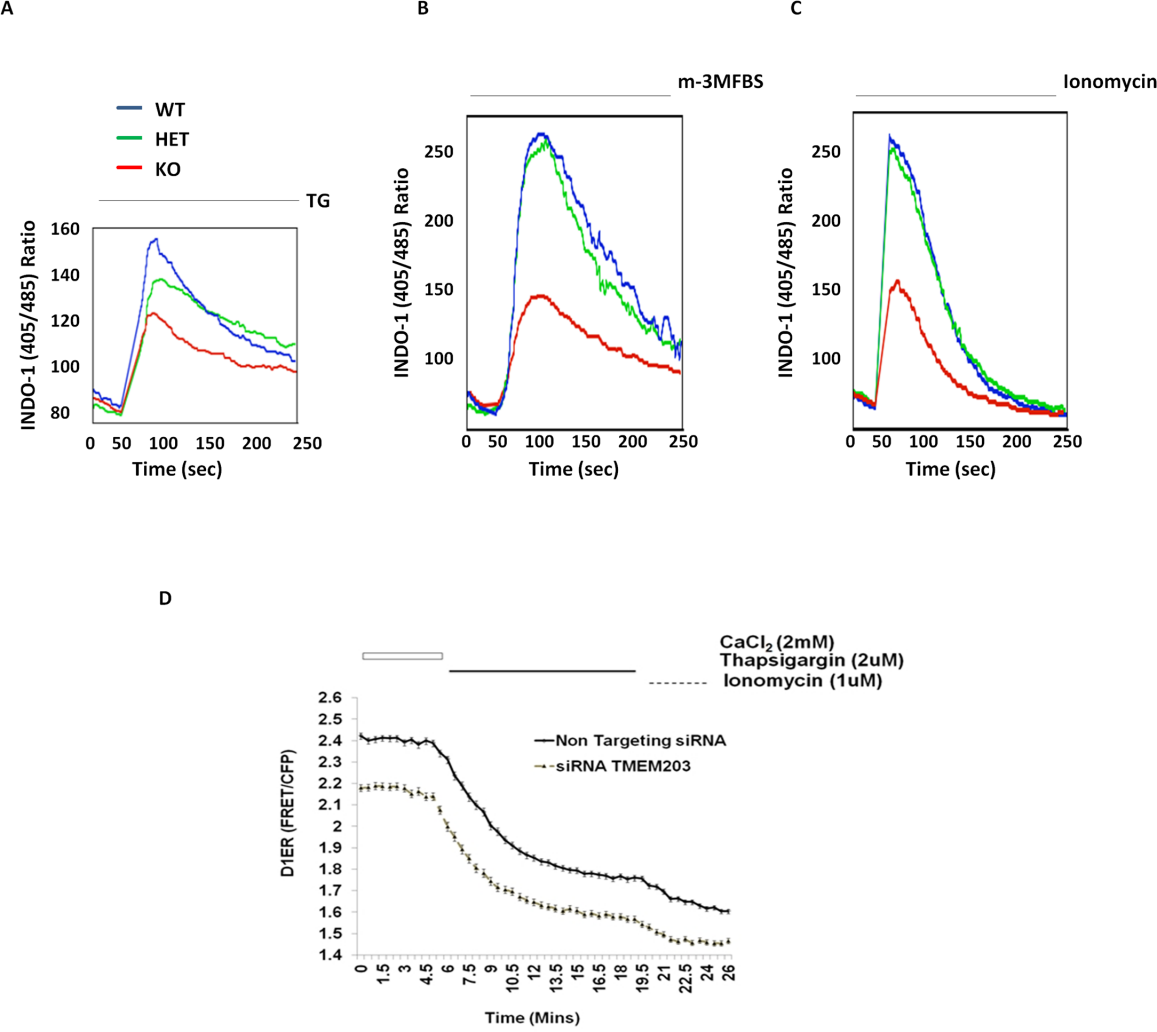
Altered calcium homeostasis in Tmem203 deficient Mouse Embryonic Fibroblast cells. (A) Cytoplasmic calcium flux were measured by flow cytometry from MEF cells derived from Tmem203—WT (Blue), HET (Green) and null (Red) mice. Treatment with 1 μM TG and EGTA in calcium free buffer showing ER-released calcium flux. Data are representative of at least 3 experiments from multiple MEF derived from littermates. (B) As described in (A), the calcium flux were measured upon treatment with 50 μM m-3M3FBS. (C) As described in (A), the calcium flux were measured upon treatment with 5 μM Ionomycin. (D) Single cell fluorescent microscopy based direct ER calcium measurements in D1ER-HEK293 cells transfected with non-targeting or TMEM203 specific siRNA. Basal ER calcium levels and ER calcium release upon TG treatment was monitored in siRNA or control siRNA transfected D1ER-HEK293 cells. The measurements showed reduced calcium levels in TMEM203 knock down cells but similar TG induced calcium leak kinetics. [Mean; +/- SE; n = 47 (non targeting siRNA) & n = 54 (TMEM203 siRNA)].

TMEM203 deficient mice were created (See S4 Fig and Methods) and ER calcium levels in Tmem203 deficient and wild type (WT) MEF cells were compared. Surprisingly, TG-induced total calcium release from the ER was substantially lower in *Tmem203* null MEF cells as compared to Het or WT MEFs (Fig 3A). Treatment with m-3mFBS, a PLC agonist which depletes ER by the activation of the IP3R, or ionomycin, also produced much lower calcium release from the *Tmem203* null MEF cells (Fig 3B and 3C). These data suggest that TMEM203 deficiency also resulted in lower ER calcium stores. The role of TMEM203 on ER calcium stores in human HEK293 cells was also examined by measuring ER calcium stores directly using the D1ER calcium sensor after siRNA knockdown of TMEM203. As shown in Fig 3D, TMEM203 siRNA treatment significantly lowered basal calcium levels. Treatment with TG further reduced ER calcium with similar kinetics and magnitude in TMEM203 and control siRNA treated cells suggesting that the steady state levels of ER calcium were reduced by TMEM203 inhibition but SERCA refilling was unaffected. Inhibition of TMEM203 expression by siRNA was confirmed by QPCR and the extent of reduction of TMEM203 expression with multiple siRNAs correlated well with the observed reduced constitutive ER calcium levels (data not shown).

Further, we looked at the expression of calcium/NFAT dependent genes—calreticulin (*Carl*) and calcitonin receptor (*Calcr*) [41,42] upon stimulation with TG or Ionomycin in MEF cells. In response to ER calcium depletion by TG or Ionomycin treatment, the expression of *Carl* and *Calcr* were induced as previously reported. Induction of *Carl* and *Calcr* were significantly reduced in *Tmem203* null MEF cells as compared to WT-MEF cells (S5 Fig) indicating that Tmem203 affects store depletion-induced calcium/NFAT dependent gene expression.

### *Tmem203* deficient male mice are infertile

Tmem203 null male and female mice were born at normal Mendelian frequency and showed no gross morphologic alterations, except for a ~10% reduction in weight. While heterozygotes were fully fertile, homozygous mating pairs produced no offspring despite the presence of copulatory plugs. Uninterrupted mating between mice of different genotypes were followed for over a period of 17 weeks (Table 1) to determine if one or both sexes were infertile. The *Tmem203* null male mice failed to produce any litter during the duration of the experiments. Female *Tmem203* null mice were fertile although the total numbers of litters (12 litters) were lower than that produced by WT or heterozygous females (which produced 22 and 23 litters, respectively).

**Table 1:** Tmem203 null male mice are sterile.

Male	Female	Mating pairs	Litters	Pups/litter	Litter/pair
tmem203 ^-/-^	tmem203 ^+/+^	6	0	0	0
tmem203 ^+/+^	tmem203 ^-/-^	5	12	5.75	2.4
tmem203 ^-/+^	tmem203 ^+/+^	5	22	7.63	4.4
tmem203 ^+/+^	tmem203 ^-/+^	5	20	8.05	4
tmem203 ^+/+^	tmem203 ^+/+^	5	23	6.86	4.6

Uninterrupted mating was performed between sexually mature (aged 8 weeks old) *Tmem203* null (tmem203 ^-/-^), heterozygous (tmem203 ^-/+^) and wild type (tmem203 ^+/+^) mice for 17 weeks. All litters and litter sizes were recorded for each mating pair.

Tmem203 expression, determined by quantitative real-time PCR from various tissue RNAs, was most abundant in testes (S6 Fig), consistent with a role for Tmem203 in male germ cell development or function. Absolute weight of testes, and mean testes/brain weight ratios at 24-weeks of age were significantly reduced in Tmem203 mutant mice compared to WT littermates (S7 Fig). The prostate gland, seminal vesicles and epididymides appeared normal in *Tmem203* null mice by light microscopy (data not shown). Further, the serum levels of Testosterone, follicle stimulating hormone and luteinizing hormone (LH) in wild type and *Tmem203* null mice were comparable (data not shown).

### *Tmem203* is essential for spermiogenesis

**Fig 4:**
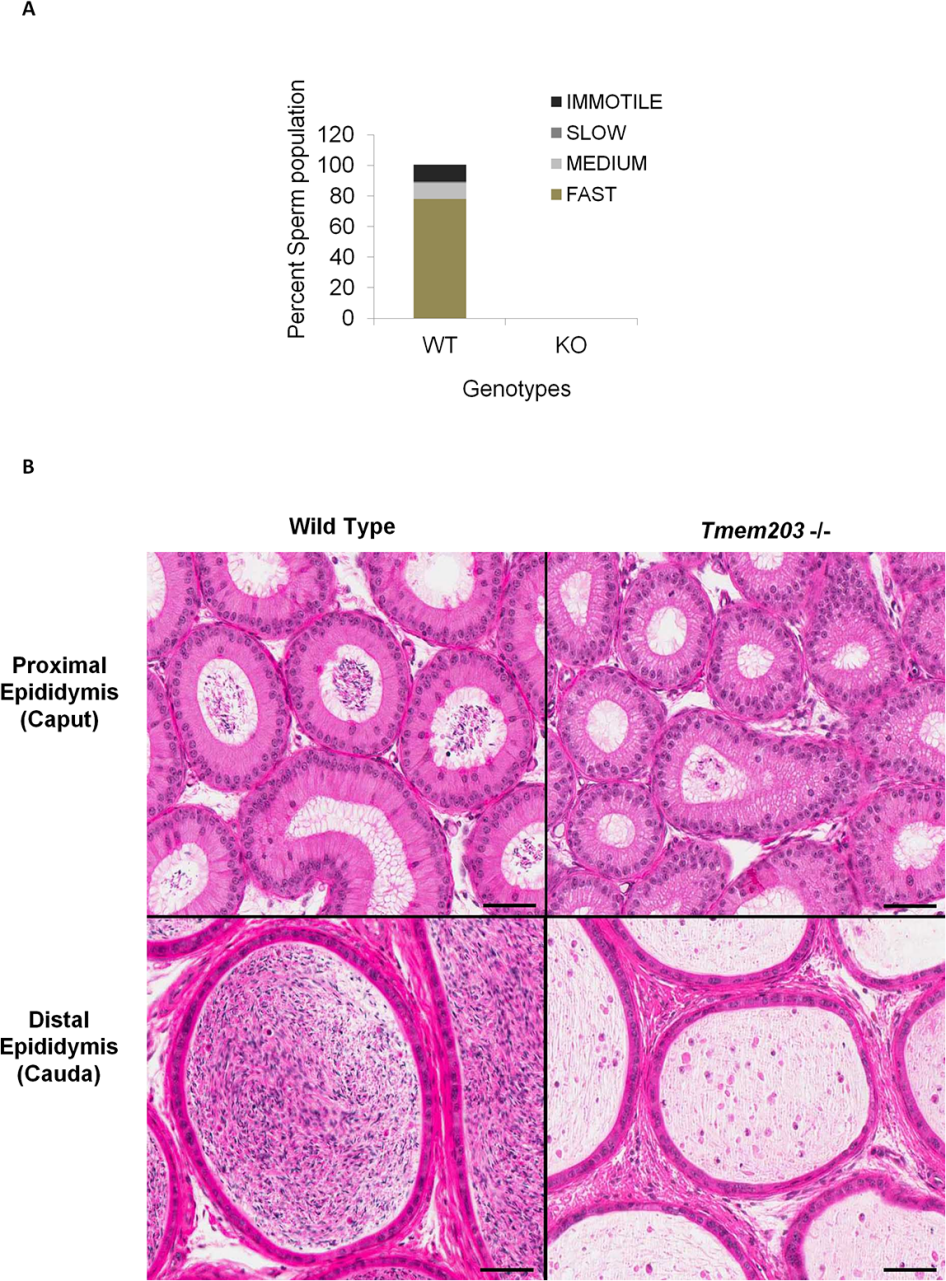
*Tmem203* null mice completely lack mature spermatozoa in epididymis. (A) Computer assisted sperm analyzer based analysis of epididymis preparations from wild type and *Tmem203* null mice showed complete absence (*) of mature spermatozoa in *Tmem203* null mice. Data is representative of two independent experiments. (B) Representative photomicrographs illustrating hematoxylin and eosin (H&E)-stained sections of proximal epididymis (caput; upper left and right panels) and distal epididymis (cauda; lower left and right panels) from a 48-week-old wild type mouse (upper and lower left panels) and from a 48-week-old *Tmem203* null mice (upper and lower right panels). Note the complete absence of mature spermatozoa in the epididymis of the *Tmem203* null mice compared to the wild type mice in which numerous mature spermatozoa are observed; tubular lumina of the epididymis from *Tmem203* null mice contains eosinophilic proteinaceous material mixed with cellular debris. Scale bars = 50 μm.

Tmem203 deficient mice lacked mature spermatozoa (complete azoospermia) as detected by a computer assisted sperm analyzer (CASA: Fig 4A). Histologic evaluation of the epididymides harvested from either 38-week-old or 48-week-old male Tmem203 deficient mice also showed a complete lack of mature spermatozoa in the epididymides (azoospermia) compared to wild type mice (Fig 4B). No abnormalities were seen in *Tmem203* heterozygous mice (data not shown).

**Fig 5:**
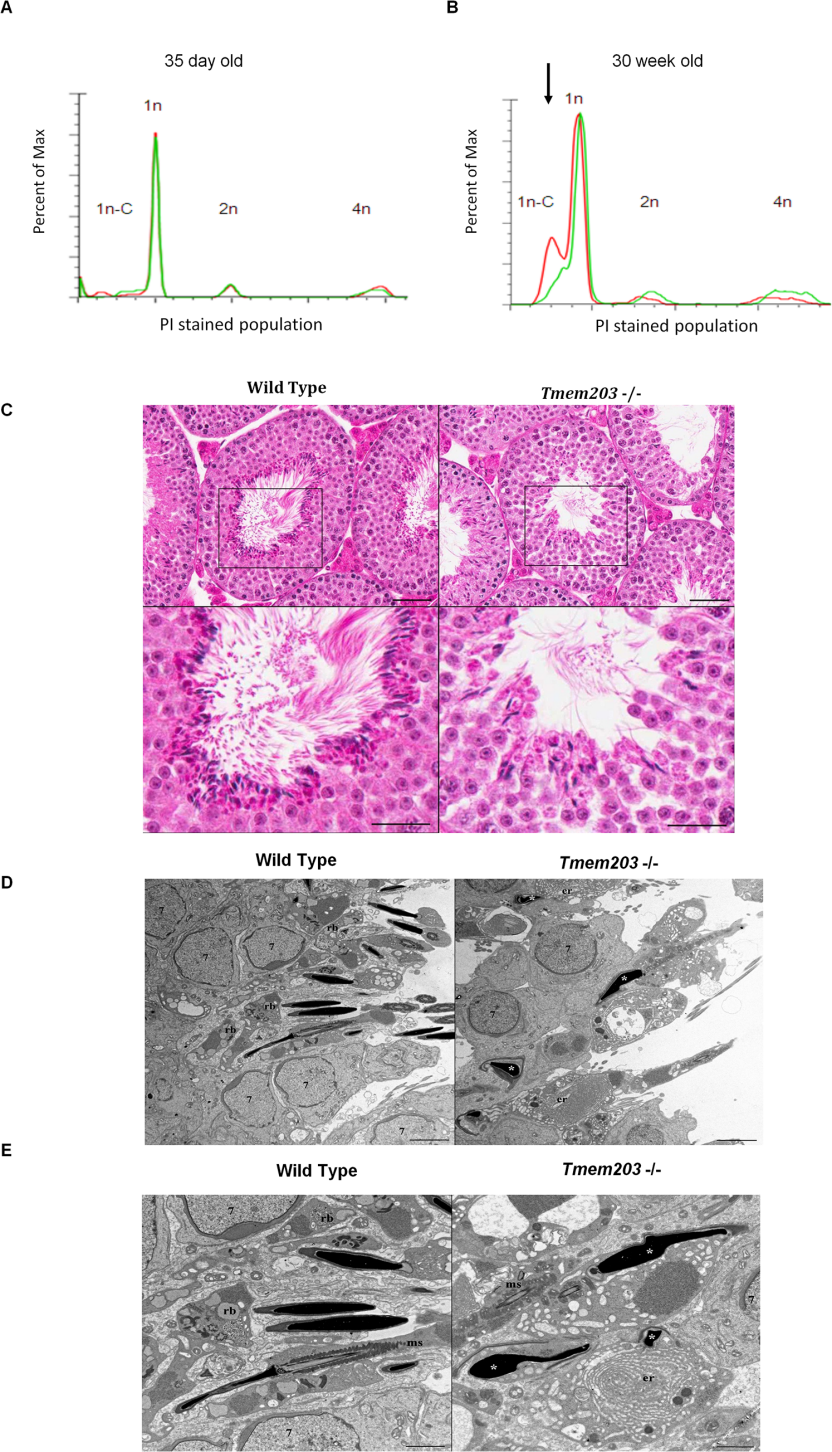
*Tmem203* null mice exhibit a disruption of spermiogenesis. (A-B) Propidium iodide based DNA flow cytometry analysis of testicular cell suspensions from wild-type (red tracer) and *Tmem*203 null mice (green tracer) at 35 day (A) or 30 week (B) (*n =* 2 or 3 for each genotype). Arrows highlight the differences between the wild-type and *Tmem203* null samples. Abbreviations: haploid-condensed (1n-C)-elongated spermatids; haploid (1n) round spermatids; diploid (2n)—Sertoli cells, spermatogonia; S-ph, spermatogonia synthesizing DNA and the tetraploid (4n)—pachytene spermatocytes and G2 spermatogonia (C) Representative photomicrographs illustrating hematoxylin and eosin (H&E) stained sections of Stage VII seminiferous tubules from a 48-week-old wild type mouse (left panels) and from a 48-week-old cMAC knockout mouse (right panels). Compared to the seminiferous tubule from the wild type mouse (left panels) the predominant morphological changes observed in the seminiferous tubule of the cMAC knockout mouse (right panels) are characterized by an overall subtle, relative reduction in numbers of late stage post-meiotic spermatids (steps 9–16), degenerative intracytoplasmic vacuolar changes most prominent in step 16 spermatids and complete lack of spermiation (disengagement of step 16 spermatozoa from the Sertoli cell and release into the tubular lumen). Lower panels illustrate higher magnification of areas enclosed by square boxes in the upper left and right panels. Scale bars = 50 μm (upper panels) and 25 μm (lower panels). (D-E) Representative transmission electron micrographs of Stage VII seminiferous tubules from a 32-week-old wild type mouse (left panels) and from a 32-week-old *Tmem203* null mouse (right panels). Labeled are step 7 spermatocytes (7), residual bodies (rb), endoplasmic reticulum (er) and degenerate, misshapen spermatid heads (asterisks) mitochondrial sheath (ms) surrounding the outer dense fibers, axoneme and axoneme complex of microtubules. Phagocytosis by Sertoli cells of degenerate spermatids is illustrated in both the top and bottom panel on the right for the *Tmem203* null mouse. Residual bodies (rb) contain dense aggregations of RNA, lipid, clear vesicles, multivesicular bodies and other organelles. Scale bars = 5.0 μm (for D),2.0 μm (for E).

To determine the nature of azoospermia in *Tmem203* null mice, spermatogenesis was examined in detail. We first evaluated the distribution of propidium iodide stained testicular cells from 35 day old or 30 week old *Tmem203* null and wild type mice. DNA staining during the first wave of spermiogenesis at day 35 was indistinguishable from WT mice (Fig 5A). In contrast, at 30 weeks old, there was a large reduction of the PI stained population of Tmem203 deficient spermatocytes corresponding to the post-meiotic condensed haploid population (1n-C) (Fig 5B). Peaks representing the other developmental stages of spermatogenic cells and progression of spermatogenesis till elongated spermatids were comparable in *Tmem203* null and wild type samples. Thus FACs analysis suggests that meiosis appears to be normal but Tmem203 deficient spermiogenesis fails beginning by the stage of nuclear condensation.

Microscopic evaluation of the testes harvested from either 38-week-old or 48-week-old male mice showed prominent defective spermiogenesis in Tmem203 deficient mice, despite a relatively normal overall structural appearance and organization of the seminiferous tubular epithelium (Fig 5C). The predominant morphological changes observed in the seminiferous tubules of *Tmem203* null mice were characterized by: a) an overall but subtle reduction in numbers of late stage post-meiotic spermatids (steps 9–16) in the stages of the cycle of spermatogenesis in which progressive elongation and condensation of the nucleus occurs [30,31], b) degenerative intra-cytoplasmic vacuolar changes most prominent in step 16 spermatids, c) complete lack of spermiation (disengagement of step 16 spermatozoa from the Sertoli cell and release into the tubular lumen), and d) retention of step 16 spermatids in stage VIII seminiferous tubules. Morphological abnormalities were not observed microscopically in *Tmem203* null mice in non-spermatogenic cells including Sertoli cells and interstitial Leydig cells. In *Tmem203* null mice, atrophy of individual seminiferous tubules was occasionally observed multifocally scattered randomly throughout testes (data not shown).

Transmission electron microscopy performed on testes harvested from 32-week-old Tmem203 null mice and age-matched wild type mice confirmed changes observed by bright field light microscopy including degenerative intra-cytoplasmic vacuolar changes as well defective spermiation. In addition, ultra-structural evaluation of the testes from Tmem203 null mice revealed abnormalities associated with the shape of the head of individual elongated spermatids (teratozoospermia) despite apparent normal condensation of chromatin and morphology of the acrosome (Fig 5D and 5E). Numerous, large, ovoid phagocytic structures which contained degenerate and fragmented elongated spermatids as well as remnants of elongated spermatid tail fragments were often observed throughout all levels of the seminiferous tubule; in Stage VII tubules these structures were most commonly observed near the lumina of seminiferous tubules (Fig 5D and 5E). Abundant amounts of intra-cytoplasmic material which ultra-structurally resembled endoplasmic reticulum were often observed in association with ovoid phagocytic structures. Despite degenerative changes associated with the head of elongated spermatids in Tmem203 null mice, the cytoskeletal components of the connecting, middle, principle and end pieces of the flagellum of individual mature elongated spermatids appeared to be ultra-structurally normal including the axoneme, axoneme complex of microtubules (two central microtubules surrounded by nine microtubule doublets) and fibrous sheath. Individual mitochondria helically wrapped around the outer dense fibers in the middle piece of the sperm tail (mitochondrial sheath) associated with step 15 spermatids in Stage VI seminiferous tubules and step 16 spermatids in Stage VII seminiferous tubules also appeared to be ultra-structurally normal (Fig 5D and 5E). To summarize, spermatogenesis defects appeared post-meiotically in Tmem203 deficient mice resulting in a severe reduction of late stage spermatids, appearance of only abnormal condensed haploid spermatocytes that fail to undergo spermiation.

### Differential gene expression in *Tmem203* null testes

**Fig 6:**
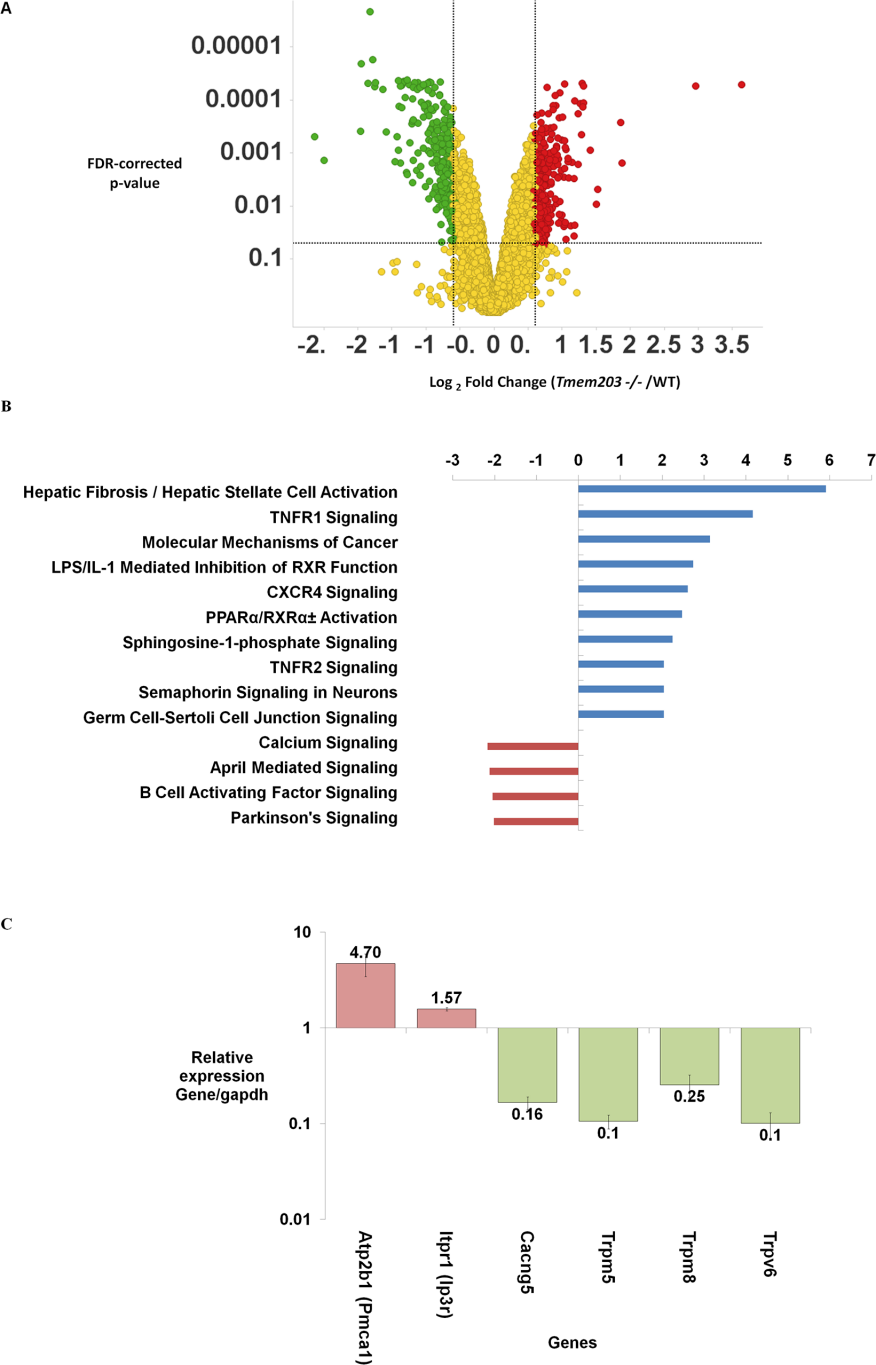
Gene expression profiling in Tmem203 null mouse testes indicates aberrant expression of key calcium channels and pumps. (A) A spotfire based visualization of differential gene expression displaying fold changes in gene expression versus FDR corrected P value obtained from a microarray based RNA expression analysis from a set of five *Tmem203* null and wild type mice. (B) Ingenuity based pathways enrichment of differentially expressed genes in *Tmem203* null mouse testes. For the analysis the pathways showing a significant change with a p value of >2 were considered. (C) Quantitative real time PCR analysis of RNA obtained from WT and *Tmem203* null mice testes for genes differentially expressed in the calcium signaling pathway selected from (B). Expression level of mentioned genes in *Tmem203* null mice testes relative to WT testes expression level after normalization to *Gapdh*. Data represents 4 replicates (+/- Std Dev) and validated in 2 or more RNA preparations from *Tmem203* null and WT testes. *—Transcript not detected in *Tmem203* null mice testes.

Expression profiling studies were carried out to identify potential molecular consequences of loss of Tmem203. We compared the expression profiles as described in the methods for testes and skeletal muscle, the highest and lowest sites of Tmem203 expression levels as seen in the tested tissues (S6 Fig). The expression profile comparisons between *Tmem203* wild type and null mice showed significant changes in testes (Fig 6A) and little or no significant alterations in skeletal muscle (data not shown). 163 and 129 known genes were found to be increased or decreased by > 1.5 fold (S2 and S3 Tables). Differentially expressed genes were analyzed for pathway enrichment using Ingenuity Pathway Analysis (IPA) (Fig 6B). The most significantly up-regulated pathways were those involved in fibrosis and inflammation. It is unclear if these changes were due directly to *Tmem203* deletion or secondary responses due to altered development of the testes. Interestingly, the most significantly down-regulated pathway was that composed of proteins annotated to regulate intracellular calcium levels. This set of genes included increased expression of inositol 1, 4, 5-triphosphate receptor 1 (Ip3r1) and ATPase, Ca^++^ transporting, plasma membrane 1 (Atp2b1 / Pmca1) RNAs and reduction ofTrpv6, Trpm5 and Trpm8 calcium channel RNAs in Tmem203 deficient testes. The differential expression of these genes was further validated by real-time quantitative RT PCR (Fig 6C). The differential expression of channels and transporters suggest that Tmem203 deficient spermatogenic cells would have altered calcium handling capacity. It should also be noted that the IPA also suggested a significant up-regulation in Germ Cell-Sertoli Cell Junction signaling genes {Ras Homolog Family Member Q (Rhoq), P21 Protein Activated Kinase 6 & 3 (Pak6, Pak3), Rho family GTPase 3 (Rnd3), integrin, alpha 6 (Itga6), catenin (cadherin-associated protein), alpha 1 (Ctnna1), junction plakoglobin (Jup)} The up-regulation of this gene-set was not pursued further as there was no obvious relevant morphological defects seen in ultra-structural studies of testes from *Tmem203* null mice.

### Altered calcium mobilization in Tmem203 null testicular cells

**Fig 7:**
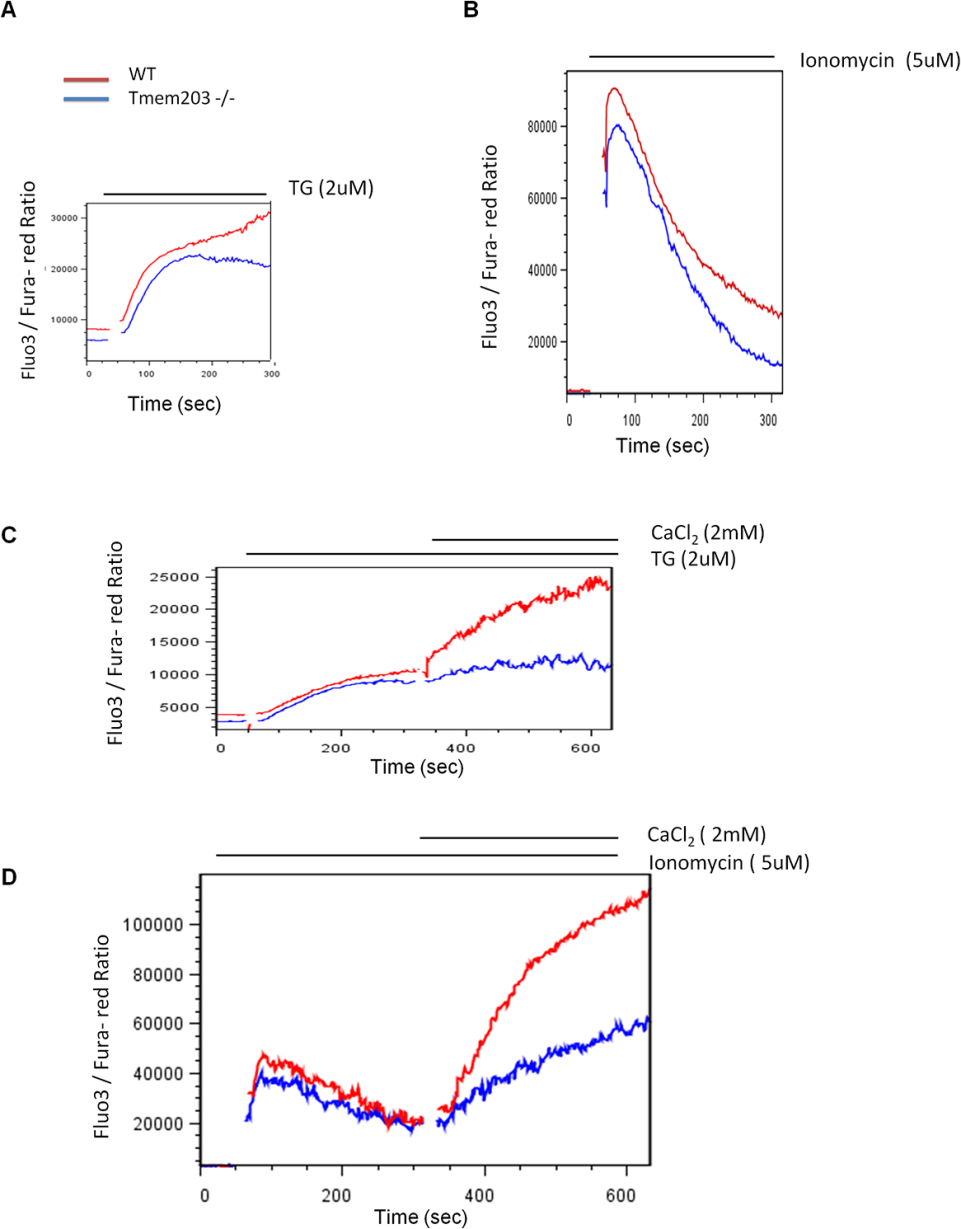
Intracellular store calcium flux and store operated calcium entry kinetics in testicular cells from WT and *Tmem203* null mice. Flou3 and Fura red loaded testicular cells prepared from WT and *Tmem203* null mice were analyzed by flow cytometry to follow cytosolic calcium kinetics in the gated predominately round spermatids population. Intracellular store calcium flux was measured by recording Flou3 calcium bound to Fura red calcium free ratio in the presence of 1mM EGTA in response to SERCA inhibitor- Thapsigargin (A); Calcium ionophore—Ionomycin (B); Similarly the store operated calcium entry kinetics was followed in WT and *Tmem203* null testicular round spermatids gated population by depleting the stores by Thapsigargin (C) or Ionomycin (D) followed with addition of 2mM CaCl_2_.

Single cell suspensions of testes from WT and *Tmem203* null mice were prepared and ratiometric calcium measurements in equivalent gated populations consisting chiefly of round spermatids [36] were performed using flow cytometry. Calcium flux was measured in response to release of intracellular calcium stores before (Fig 7A and 7B) and after addition of extracellular calcium (Fig 7C and 7D). The *Tmem203* null cells exhibited modestly lower basal cytoplasmic calcium levels at rest. The rises in intracellular calcium immediately after store depletion were modestly lower than WT mice. *Tmem203* null cells exhibited a reduction of cytosolic calcium after store depletion by TG in contrast to WT cells which showed a continuous rise in cytoplasmic calcium. *Tmem203* null cells showed a more pronounced reduction of cytosolic calcium after the peak levels induced by ionomycin (Fig 7B). After addition of calcium to the media after store depletion by either TG or Ionomycin, *Tmem203* null cells exhibited significantly reduced cytoplasmic calcium accumulation compared to WT cells (Fig 7C and 7D). These data are consistent with both reduced calcium uptake and increased cellular extrusion predicted by the expression profiling data which showed reduced expression of calcium import channels and increased expression of the predominant cellular calcium pump. These data suggest that the defective spermiogenesis in Tmem203 deficient mice is due to alterations in calcium homeostasis.

## Discussion

This study describes the identification of TMEM203 as a novel regulator of ER calcium levels. TMEM203 was identified here by its ability upon overexpression to mediate calcium dependent nuclear relocalization of the CRCT1 and NFAT transcription factors. TMEM203 is an evolutionary conserved resident ER protein that bears little amino acid sequence similarity to other proteins. TMEM203 was required to maintain normal ER calcium levels in a variety of cell types. Mice lacking Tmem203 expression were viable though male knockout mice were infertile and exhibited a severe block in spermiogenesis and spermiation which were also accompanied by altered accumulation of cellular calcium. Thus Tmem203 represents an evolutionarily conserved regulator of intracellular calcium homeostasis and provides a causal link between intracellular calcium regulation and spermiogenesis.

The ability of TMEM203 to induce nuclear translocation of NFAT and CRTC1 required activation of calcineurin likely following severe loss of ER calcium stores and activation of store operated calcium entry (SOCE). The mechanism by which TMEM203 overexpression releases ER calcium stores is not clear. Exogenously expressed TMEM203 was localized to the ER (Fig 2A) and was found in a complex with a number of calcium regulatory proteins, including STIM1, SERCA2 and IP3R (Fig 2B). The activity of TMEM203 could be explained by regulation of one or more complexed proteins, for example by either inhibition of calcium refilling via SERCA2 or activation of the IP3R. Alternatively, overexpressed TMEM203 could form a pore, increasing the leak of calcium stores as has been suggested for other overexpressed proteins such as BCL-2, the Bax inhibitory protein-1 or presinillins [43,44].

TMEM203 depletion, either via siRNA or through gene disruption also lowered ER calcium stores (Fig 3A–3D). Thus, at least in some cell types, TMEM203 appeared to play an essential role in maintaining ER calcium. It is not clear why overexpression and loss of TMEM203 expression both resulted in reduction of ER calcium stores. One explanation is that overexpression of TMEM203 may act in a dominant negative fashion, perhaps titrating out either endogenous TMEM203 or TMEM203 interacting proteins. However, it is worth noting that the effects of TMEM203 inhibition and overexpression are distinct in some ways. For instance, while overexpression of TMEM203 appears to elevate the basal cytosolic calcium levels and activate NFAT and CRTC1 (Fig 1A–1D), loss of Tmem203 has an inhibitory effect on NFAT dependent gene expression in mouse fibroblasts (S5 Fig). These observations suggest that overexpression and reduction of TMEM203 have distinct physiologic affects and that TMEM203 likely regulates ER calcium via multiple mechanisms.

In this regard it is worth noting that other proteins, notably of the BCL-2 family, have been reported to have bidirectional/opposing effects on ER calcium stores. For example, overexpression of BCL-2 lowers ER calcium stores by several reported mechanisms including acting as a leak channel, inhibiting refilling by SERCA and by enhancing activation and calcium release by the IP3R [45]. Conversely BCL-2 has also been suggested to act as an inhibitor of the IP3R, preventing calcium release during stress. Similarly, BAX overexpression has been reported to reduce ER calcium stores [46], whereas inactivation of BAX and BAK also reduces ER calcium levels through enhanced calcium leak and IP3R activation [47,48]. The precise mechanism by which TMEM203 affects ER calcium stores will await further studies including the biochemical purification and characterization of TMEM203 and associated protein complexes.

Tmem203 deficient male mice were infertile with a failure to complete spermatogenesis and spermiogenesis. At present, morphologic analysis does not pinpoint a single aspect of spermatogenesis affected by TMEM203 deficiency. Haploid spermatids appear to be reduced in numbers and spermiogenesis fails after nuclear condensation, resulting in a large reduction in elongated spermatids, and accumulation of small numbers of degenerative malformed sperm heads which fail to undergo spermiation (Fig 5A–5E).While the exact cause of spermiogenic failure is not clear, calcium homeostasis in testicular cells was clearly defective; suggesting that defective spermiogenesis may be due to altered handling of calcium stores (Fig 7A–7D). Expression profiling experiments demonstrated significantly altered expression of calcium pumps and channels in Tmem203 deficient mice (Fig 6C). The increased expression of the main calcium extrusion pump, Pmca1 and decreased expression of multiple import channels seems likely to contribute to modestly reduced basal calcium levels and highly reduced retention of calcium after SOCE seen *in vitro* with primary spermatocytes. The altered expression of calcium pumps and channels might be due to either an attempt by cells to compensate for loss of Tmem203 or indirectly due to developmental defects. It is worth noting that loss of Bcl2 also appears to result in a dramatically increased rate of calcium extrusion in pancreatic acinar cells due to increased function of the Pmca calcium pump [49].

A clear role for calcium stores in spermiogenesis has not been directly described although a number of genes regulated by calcium have been shown to be essential for sperm development. *C elegans* lacking expression of mammalian homologs of calreticulin (Crt-1)—the calcium binding chaperone protein or calcineurin (Cna or Cnb)—the calcium dependent phosphatase are sterile due to defects in sperm development in addition to oocyte development and fertilization [50,51]

A similar spermiogenesis defect as in *Tmem203* null mice was described with Calcium and integrin binding 1 (CIB1)/calmyrin null mice [52]. CIB1 is a homolog of Calmodulin and Calcineurin B and regulates calcium dependent signaling events [53]. It will be of interest to determine if CIB1 and Tmem203 act in a common pathway involved in calcium/calcineurin signaling and spermatogenesis in mammals. Finally, Bax is also required for spermiogenesis in mice [54] and as mentioned above, also modulates levels of ER calcium similar to Tmem203. The similarity in phenotypes seen in Tmem203 and Bcl2 family protein deficient mice [37] suggests that these proteins may converge on common mechanisms that govern ER calcium stores and cellular calcium handling.

The defective calcium handling in testicular cell preparations, which were predominantly haploid spermatocytes (Fig 7A–7D), suggests that Tmem203 deficiency directly affects development or survival of spermatocytes. However, it is also possible that defects Sertoli and Lydig cells could also play some or a major role in the infertility in Tmem203 deficient mice. Spermiation, the process of releasing mature sperm from the Sertoli cells into the seminiferous tubule was completely absent in Tmem203 deficient mice, while it is possible that Tmem203 is directly involved in spermiation, it seems likely this defect is due to the severe loss of maturing spermatids, miss-shappen post meiotic spermatids and the likely clearance of remaining malformed spermatids by Sertoli cells before spermiation. The simplest explanation for the phenotypes observed is that the defective calcium handling within spermatocytes results in developmental arrest or cell death of spermatocytes shortly after the stage of nuclear condensation.

*Tmem203* is a highly conserved gene and is absolutely requirement for fertility. Tmem203’s role in maintaining steady state ER calcium levels suggests it may modulate a variety of biological processes. Indeed, preliminary analysis suggests that TMEM203 deficiency also results in other phenotypes. TMEM203 deficient mice are about 10% smaller than WT siblings, have mild immunologic defects at least *in vitro*, and exhibit evidence for impaired neurologic function, though these phenotypes have not been sufficiently characterized for publication yet. The absolute requirement for TME203 in fertility would seem to be sufficient to explain its high level of evolutionary conservation. *Tmem203* null mice may serve as an excellent tool to understand the regulation of intracellular calcium levels and its interdependence on carefully orchestrated modulation of ER calcium stores. Tmem203’s role in spermatogenesis adds to the complexity of this fascinating process and strongly suggests a role of intracellular calcium homeostasis during mammalian spermatogenesis.

## Supporting Information

S1 Fig TMEM203 expression drives calcineurin and calcium dependent NFAT transcription factor activation. TMEM203-FLAG over-expression in 293 cells for 48 hrs induced NFAT dependent luciferase expression in a dose dependent manner, Treatment with 1uM PMA for 8 hrs enhanced TMEM203 mediated NFAT activation whereas a treatment with 5uM cyclosporine A or 10 nM SKF96365 inhibited TMEM203’s activity. Data is representative of at least 3 independent experiments (mean; +/- SD; n = 3).
(TIF)

S2 Fig TMEM203 is a predicted integral membrane protein that is highly conserved across vertebrates. (A)-Trans-membrane domain prediction of the TMEM203 sequence of using the TMHMM algorithm. TMEM203 is predicted to have 4 pass TM domains (red) with 3 loops and both N terminal and C terminal (green) facing the same side. (B)- TMEM203 amino acid sequences from various organisms were analyzed for conservation using PRALINE multiple sequence alignment tool. (http://www.ibi.vu.nl/programs/pralinewww/). The following TMEM203 protein sequences were retrieved from NCBI—*Homo sapiens* NP_444273; *Mus musculus* NP_796318; *Xenopus laevis* NP_001085810; *Macaca mulatta* NP_001248131; *Gallus gallus* XP_001234196; *Danio rerio* NP_001002519.
(TIF)

S3 Fig TMEM203 complexes with STIM1. Western analysis of complexes immune-precipitated with endogenous STIM1 from HEK293 cells with indicated antibodies shows specific interaction with overexpressed TMEM203-FLAG. (Representative of atleast 2 independent experiments).
(TIF)

S4 Fig *Tmem203* gene targeting strategy. (A)- As described in the methods section. WT: wild type allele; KO+neo: knockout allele with neo; KO: knockout allele without neo. B: BamHI. (B). Gene disruption was confirmed by sequencing *Tmem203* cDNA from lung tissue. The 102 bp fragment from the vector (marked in bold and underlined) disrupts the adjoining *Tmem203* gene sequence in the *Tmem203* null genome. The disrupted *Tmem203* gene in the *Tmem203* null genome lacks the start codon (ATG) for *Tmem203* orf. While antibody adequate for detection of TMEM203 protein are not available, sequencing of cDNAs demonstrate that remaining tmem203 mRNA from knockout animals could only encode the last 38 amino acids of the predicted tmem 203 protein.
(TIF)

S5 Fig Altered calcium/NFAT dependent gene expression in Tmem203 deficient Mouse Embryonic Fibroblast cells. **(A)** MEF cells derived from Tmem203—WT and null mice were stimulated with 100 nM TG for 4 hrs and subsequently the expression of calcium/NFAT dependent genes—Calreticulin (Calr) or Cacitonin receptor (Caclr) were determined by quantitative real-time PCR. (Mean; +/-; SD n = 4). Significant difference in Carl expression (P value = 0.0000001) and Caclr expression (P value = 0.0000002) was observed between WT and Tmem203 null MEF cells. **(B)** As described in (A), the expression of Carl and Caclr was determined in response to stimulation with 1uM ionomycin. Significant difference in Carl expression (P value = 0.000002) and Caclr expression (P value = 0.00000004) was observed between WT and Tmem203 null MEF cells.
(TIF)

S6 Fig Tmem203 is robustly expressed in mouse testes. Quantitative real-time PCR analysis of Tmem203 transcript from various mouse tissue types showing abundant expression of Tmem203 in testes.
(TIF)

S7 Fig Body weight and Testes weight analysis of *Tmem203* null male mice. Compared to wild type control mice, *Tmem203* null mice had significantly lower (A) Mean absolute body weights; 32.93 ± 0.80 grams versus 27.66 ± 1.98 grams, respectively; p value = 0.0006. (B) Mean absolute combined left and right testes weights; 0.2216 ± 0.103 grams versus 0.1608 ± 0.0142 grams, respectively; p value < 0.0001. (C) Mean testes to body weight ratios; 0.0067 ± 0.004 versus 0.0058 ± 0.0007 grams, respectively; p value = 0.0458. (D) Mean testes to brain weight ratios; 0.4544 ± 0.0570 grams versus 0.3504 ± 0.0332 grams, respectively; p value = 0.0078.
(TIF)

S1 Table cDNAs that induce CRTC1 translocation. List of cDNAs, in addition to TPRV6 and PKA that induced translocation of CRTC1 to the nucleus in a HeLa CRTC1-eGFP expressing cell line. However, only TMEM203, could efficiently translocate TORC1 without inducing gross morphologic and/or apoptotic changes.
(DOCX)

S2 Table List of genes down-regulated in *Tmem203* null mouse testes. Only genes with Avg Log_2_ FC < -0.5 are shown.
(DOCX)

S3 Table List of genes up-regulated in *Tmem203* null mouse testes. Only genes with Avg Log_2_ FC >0.5 are shown.
(DOCX)
