# Supplementary figures and images for: TMEM203 Is a Novel Regulator of Intracellular Calcium Homeostasis and Is Required for Spermatogenesis
Source: PLoS One. 2015 May 21;10(5):e0127480. doi: 10.1371/journal.pone.0127480 (PMC4440627; doi:10.1371/journal.pone.0127480)

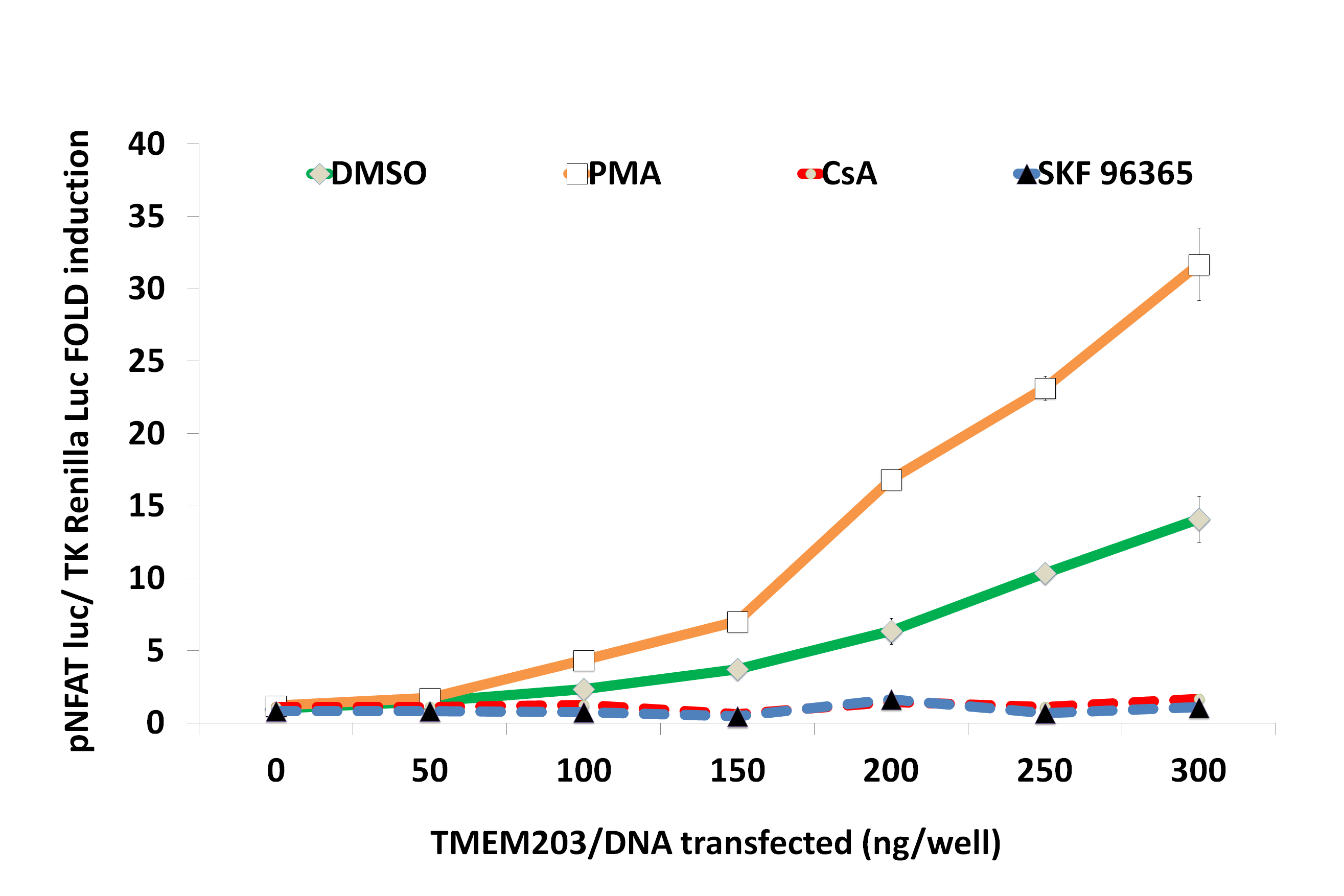

Supplement: S1 Fig — TMEM203-FLAG over-expression in 293 cells for 48 hrs induced NFAT dependent luciferase expression in a dose dependent manner, Treatment with 1uM PMA for 8 hrs enhanced TMEM203 mediated NFAT activation whereas a treatment with 5uM cyclosporine A or 10 nM SKF96365 inhibited TMEM203’s activity. Data is representative of at least 3 independent experiments (mean; +/- SD; n = 3). (TIF) [file pone.0127480.s001.tif]

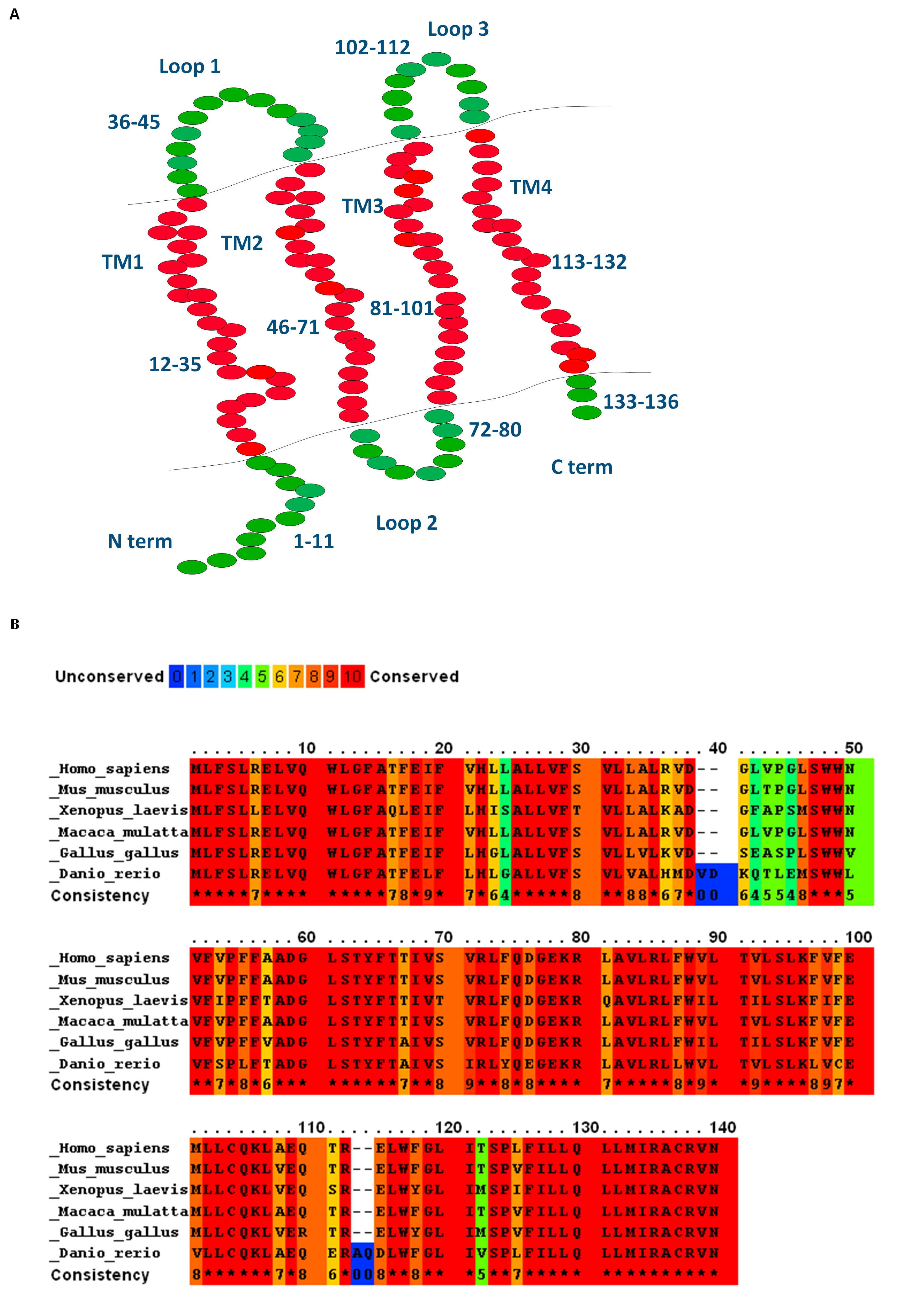

Supplement: S2 Fig — (A)-Trans-membrane domain prediction of the TMEM203 sequence of using the TMHMM algorithm. TMEM203 is predicted to have 4 pass TM domains (red) with 3 loops and both N terminal and C terminal (green) facing the same side. (B)- TMEM203 amino acid sequences from various organisms were analyzed for conservation using PRALINE multiple sequence alignment tool. (http://www.ibi.vu.nl/programs/pralinewww/). The following TMEM203 protein sequences were retrieved from NCBI—Homo sapiens NP_444273; Mus musculus NP_796318; Xenopus laevis NP_001085810; Macaca mulatta NP_001248131; Gallus gallus XP_001234196; Danio rerio NP_001002519. (TIF) [file pone.0127480.s002.tif]

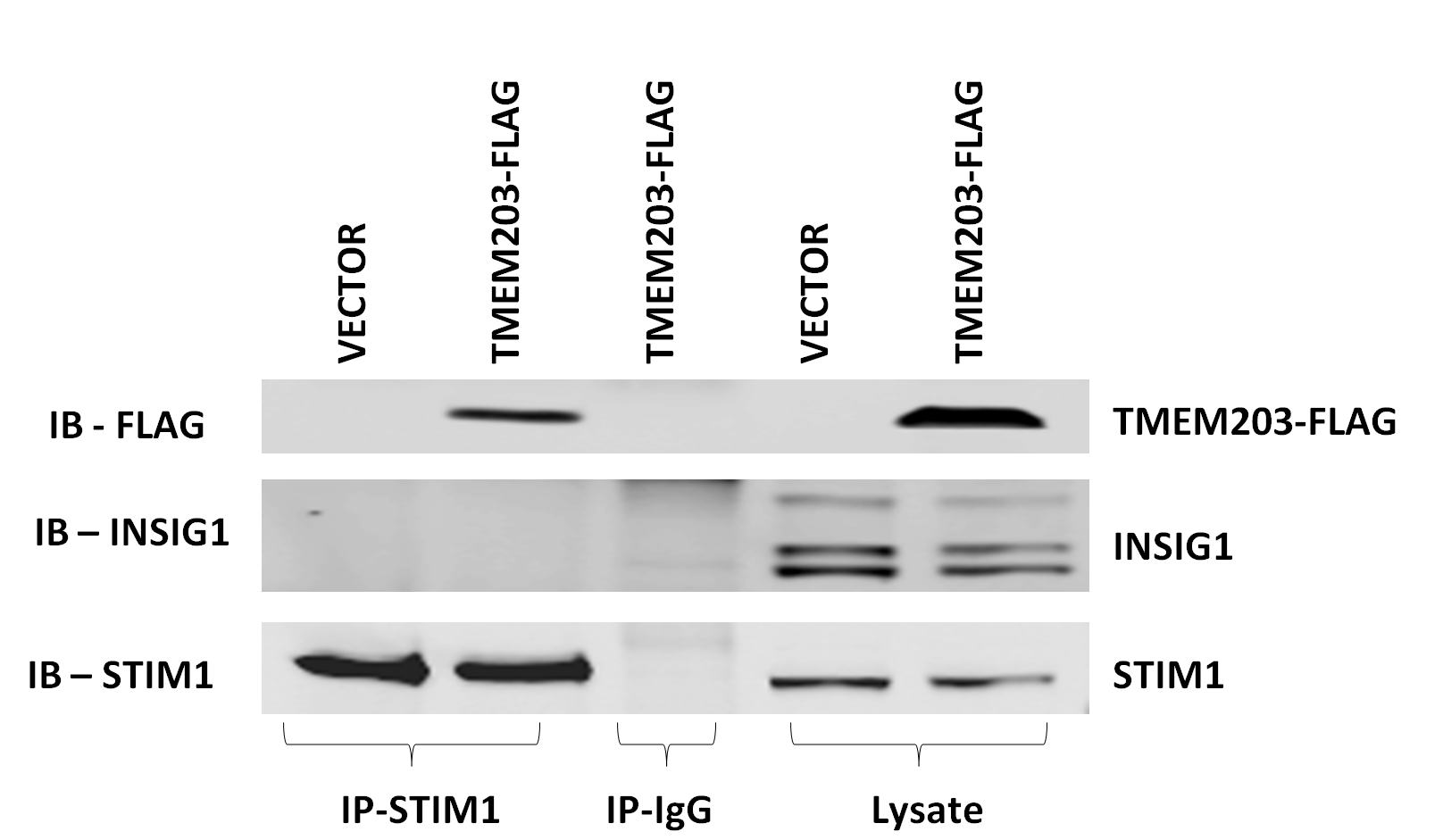

Supplement: S3 Fig — Western analysis of complexes immune-precipitated with endogenous STIM1 from HEK293 cells with indicated antibodies shows specific interaction with overexpressed TMEM203-FLAG. (Representative of atleast 2 independent experiments). (TIF) [file pone.0127480.s003.tif]

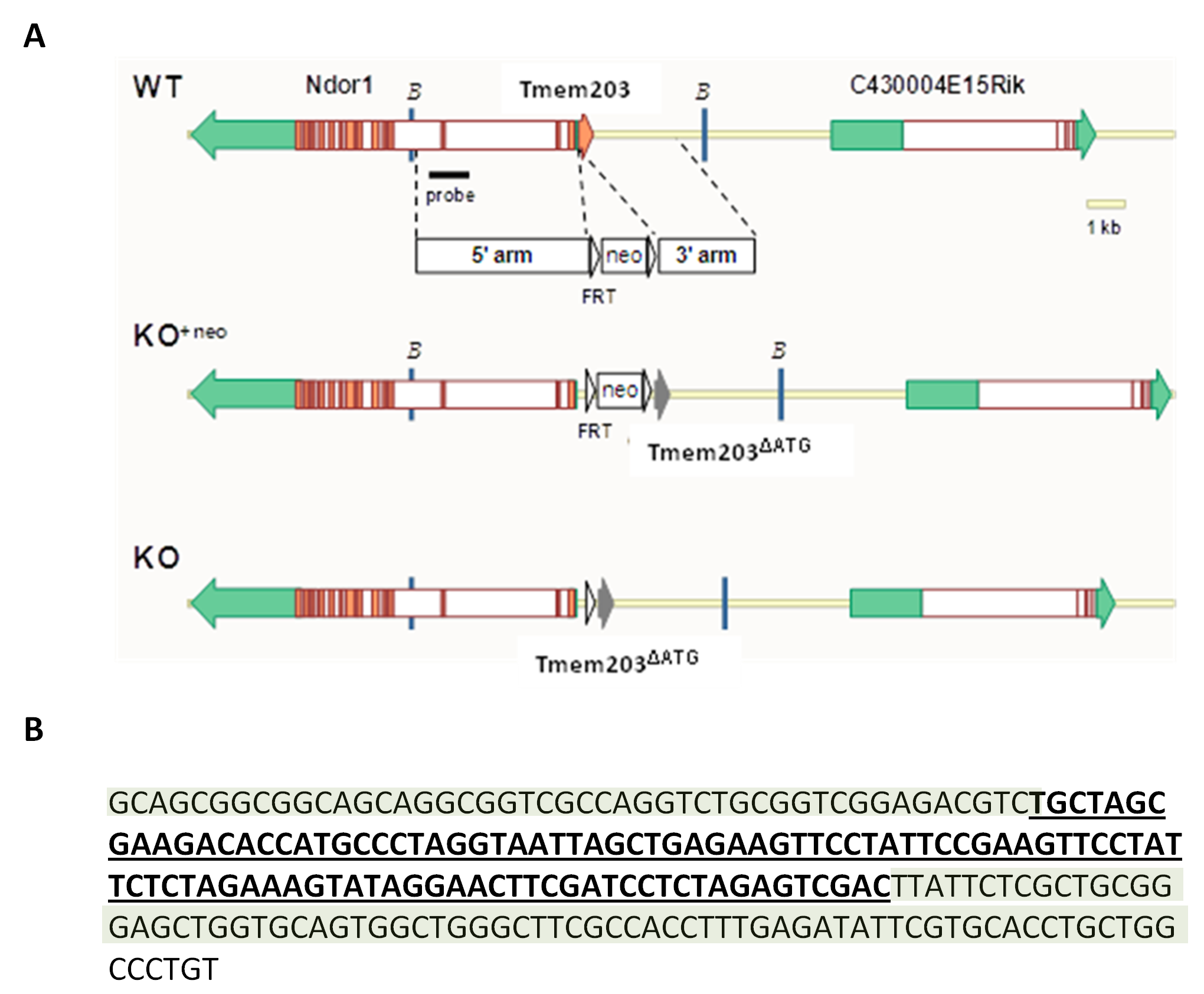

Supplement: S4 Fig — (A)- As described in the methods section. WT: wild type allele; KO+neo: knockout allele with neo; KO: knockout allele without neo. B: BamHI. (B). Gene disruption was confirmed by sequencing Tmem203 cDNA from lung tissue. The 102 bp fragment from the vector (marked in bold and underlined) disrupts the adjoining Tmem203 gene sequence in the Tmem203 null genome. The disrupted Tmem203 gene in the Tmem203 null genome lacks the start codon (ATG) for Tmem203 orf. While antibody adequate for detection of TMEM203 protein are not available, sequencing of cDNAs demonstrate that remaining tmem203 mRNA from knockout animals could only encode the last 38 amino acids of the predicted tmem 203 protein. (TIF) [file pone.0127480.s004.tif]

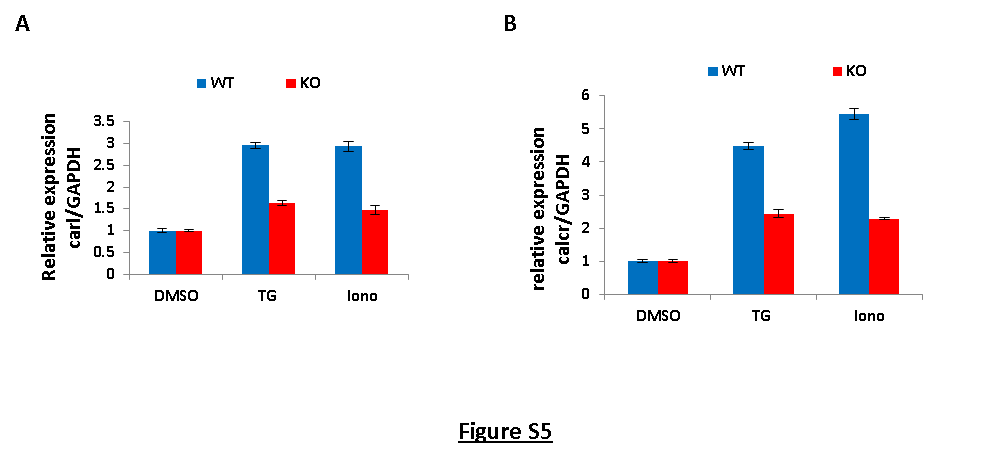

Supplement: S5 Fig — (A) MEF cells derived from Tmem203—WT and null mice were stimulated with 100 nM TG for 4 hrs and subsequently the expression of calcium/NFAT dependent genes—Calreticulin (Calr) or Cacitonin receptor (Caclr) were determined by quantitative real-time PCR. (Mean; +/-; SD n = 4). Significant difference in Carl expression (P value = 0.0000001) and Caclr expression (P value = 0.0000002) was observed between WT and Tmem203 null MEF cells. (B) As described in (A), the expression of Carl and Caclr was determined in response to stimulation with 1uM ionomycin. Significant difference in Carl expression (P value = 0.000002) and Caclr expression (P value = 0.00000004) was observed between WT and Tmem203 null MEF cells. (TIF) [file pone.0127480.s005.tif]

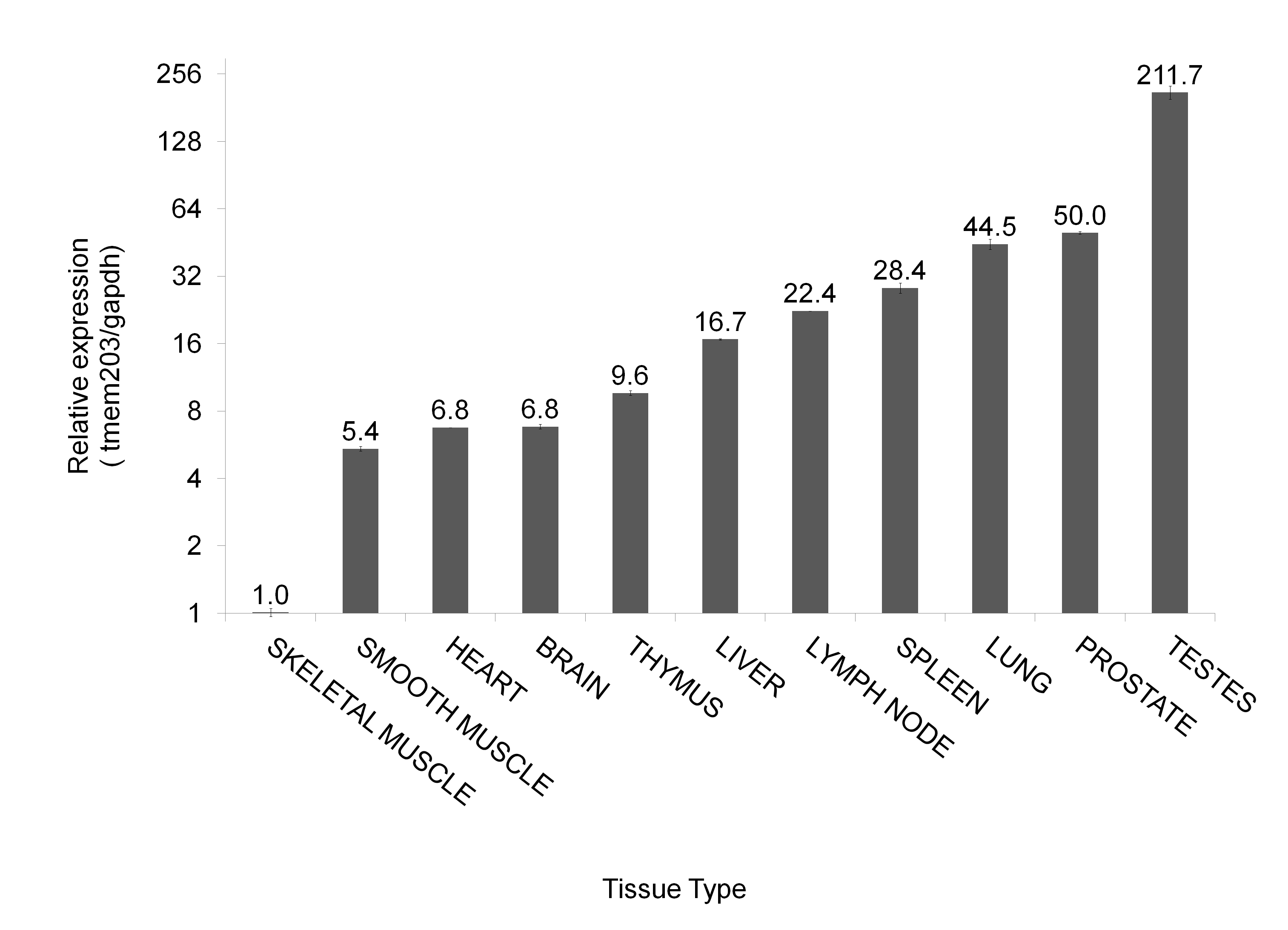

Supplement: S6 Fig — Quantitative real-time PCR analysis of Tmem203 transcript from various mouse tissue types showing abundant expression of Tmem203 in testes. (TIF) [file pone.0127480.s006.tif]

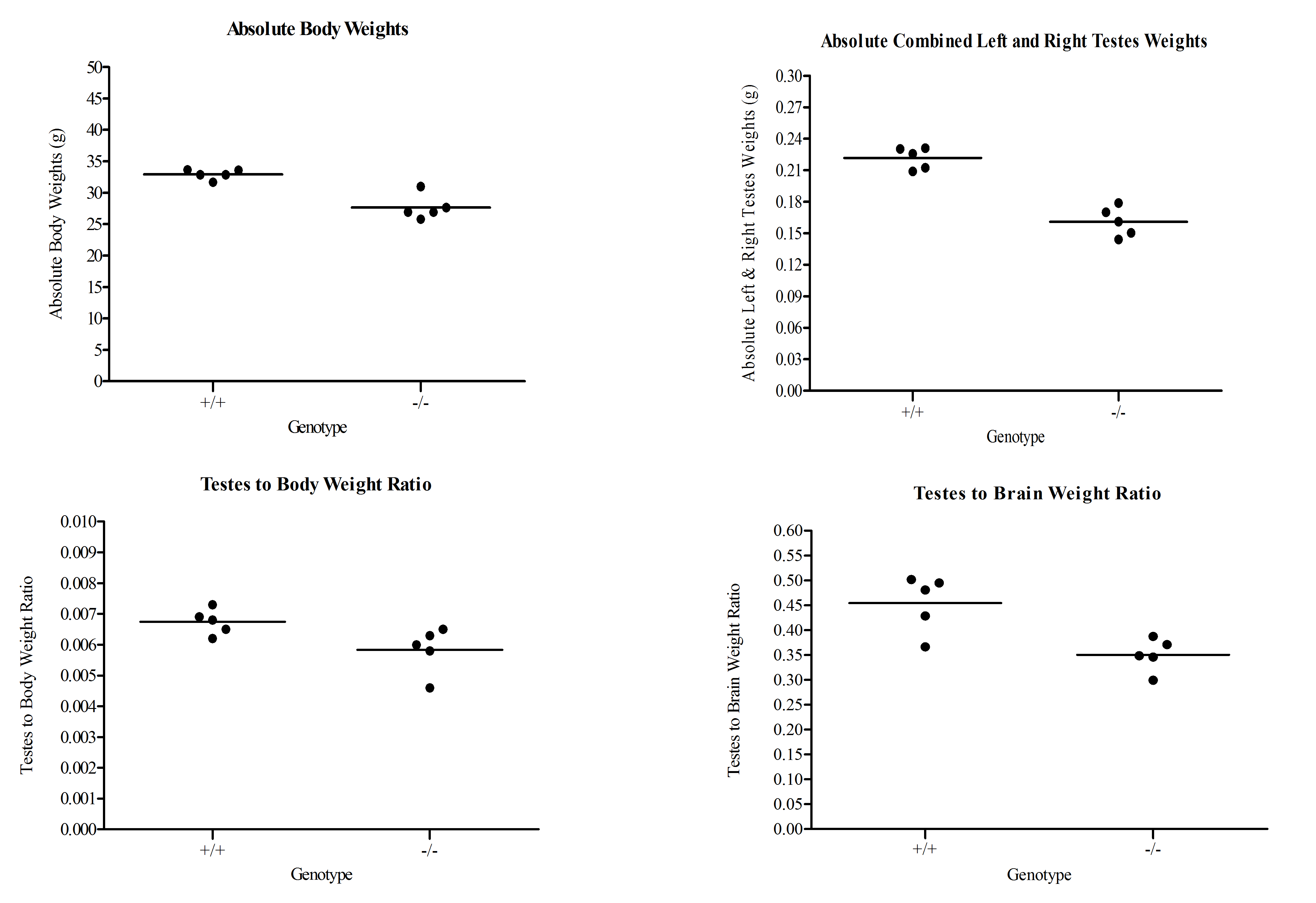

Supplement: S7 Fig — Compared to wild type control mice, Tmem203 null mice had significantly lower (A) Mean absolute body weights; 32.93 ± 0.80 grams versus 27.66 ± 1.98 grams, respectively; p value = 0.0006. (B) Mean absolute combined left and right testes weights; 0.2216 ± 0.103 grams versus 0.1608 ± 0.0142 grams, respectively; p value < 0.0001. (C) Mean testes to body weight ratios; 0.0067 ± 0.004 versus 0.0058 ± 0.0007 grams, respectively; p value = 0.0458. (D) Mean testes to brain weight ratios; 0.4544 ± 0.0570 grams versus 0.3504 ± 0.0332 grams, respectively; p value = 0.0078. (TIF) [file pone.0127480.s007.tif]
