# Supplementary material for: TMEM203 Is a Novel Regulator of Intracellular Calcium Homeostasis and Is Required for Spermatogenesis
Source: PLoS One. 2015 May 21;10(5):e0127480. doi: 10.1371/journal.pone.0127480 (PMC4440627; doi:10.1371/journal.pone.0127480)
Supplement: S1 Table — List of cDNAs, in addition to TPRV6 and PKA that induced translocation of CRTC1 to the nucleus in a HeLa CRTC1-eGFP expressing cell line. However, only TMEM203, could efficiently translocate TORC1 without inducing gross morphologic and/or apoptotic changes. (DOCX) [file pone.0127480.s008.docx]

**Supplementary Table S1**- cDNAs that induce CRTC1 translocation.

List of cDNA, in addition to TPRV6 and PKA that translocated TORC1-eGFP to the nuclei in HeLa stable line. However, only TMEM203, could efficiently translocate TORC1 without inducing gross morphologic and/or apoptotic changes

| MGC 7K designation | Annotation | Abbreviation |
| --- | --- | --- |
| 20-F3 | Mus musculus ectonucleotide pyrophosphatase/phosphodiesterase 2, mRNA (cDNA clone MGC:6665 IMAGE:3499038), complete cds | Enpp2 |
| 9-I9 | Mus musculus zinc finger protein 61, mRNA (cDNA clone MGC:11505 IMAGE:3967527), complete cds | Zfp61 |
| 9-P16 | Mus musculus receptor (calcitonin) activity modifying protein 1, mRNA (cDNA clone MGC:13748 IMAGE:4207524), complete cds | Ramp1 |
| 15-P2 | Mus musculus RIKEN cDNA C730025P13 gene, mRNA (cDNA clone MGC:31129 IMAGE:4165766), complete cds. | Tmem203 |
| 11-B13 | Mus musculus potassium inwardly-rectifying channel, subfamily J, member 15, mRNA (cDNA clone MGC:18857 IMAGE:4221516), complete cds | Kcnj15 |
| 15-P10 | Mus musculus RIKEN cDNA 1810045K07 gene, mRNA (cDNA clone MGC:31156 IMAGE:4187981), complete cds. | 1810045K07Rik |
