# Supplementary material for: TMEM203 Is a Novel Regulator of Intracellular Calcium Homeostasis and Is Required for Spermatogenesis
Source: PLoS One. 2015 May 21;10(5):e0127480. doi: 10.1371/journal.pone.0127480 (PMC4440627; doi:10.1371/journal.pone.0127480)
Supplement: S2 Table — Only genes with Avg Log2 FC < -0.5 are shown. (DOCX) [file pone.0127480.s009.docx]

**Supplementary Table S2** – List of genes down-regulated in *Tmem203* null mouse testes

| Gene Symbol | Avg Log_2_ FC | Gene Symbol | Avg Log_2_ FC | Gene Symbol | Avg Log_2_ FC | Gene Symbol | Avg Log_2_ FC |
| --- | --- | --- | --- | --- | --- | --- | --- |
| Cd164l2 | -1.93 | Nmur1 | -0.96 | Gsdmd | -0.75 | Ncs1 | -0.66 |
| Tmod4 | -1.76 | Rya3 | -0.96 | Traf1 | -0.75 | BC048679 | -0.66 |
| Klk1b5 | -1.75 | Elmo3 | -0.95 | Usp3 | -0.74 | Odf3b | -0.66 |
| Ngb | -1.75 | Fabp1 | -0.94 | Sox10 | -0.74 | Tnnc2 | -0.66 |
| Dab1 | -1.49 | 1700019G17Rik | -0.94 | 4930448F12Rik | -0.73 | Tubb2a | -0.65 |
| Gif | -1.42 | Qtrtd1 | -0.93 | Ghr | -0.73 | Aars2 | -0.65 |
| Ifrd2 | -1.41 | 4921508M14Rik | -0.92 | St8sia5 | -0.73 | Lrrc3 | -0.65 |
| Nlrc5 | -1.41 | Klk1 | -0.91 | Chac1 | -0.73 | Wipi1 | -0.64 |
| Trpm5 | -1.38 | Hhipl2 | -0.91 | Pcdh7 | -0.72 | Tbck | -0.64 |
| Ptrh1 | -1.34 | Ptp4a3 | -0.9 | Cybrd1 | -0.71 | Nfatc2 | -0.64 |
| Calml4 | -1.31 | Srgap1 | -0.88 | Six4 | -0.71 | 4933433P14Rik | -0.63 |
| Tef | -1.29 | Eomes | -0.87 | Onecut3 | -0.71 | Ccrl2 | -0.63 |
| Zim2 | -1.27 | Hpd | -0.87 | Trpm8 | -0.71 | Cplx1 | -0.63 |
| Mlkl | -1.25 | 4933406B17Rik | -0.87 | 1700007I08Rik | -0.7 | Cntfr | -0.63 |
| B4galt5 | -1.21 | Cdkn1c | -0.87 | Snx29 | -0.7 | Obfc1 | -0.63 |
| Nfe2 | -1.21 | Spns2 | -0.83 | Prr9 | -0.7 | Add2 | -0.62 |
| Kcnip2 | -1.19 | Clps | -0.83 | Grm5 | -0.7 | Dok7 | -0.62 |
| Syt13 | -1.18 | Cacng5 | -0.83 | Fgf18 | -0.7 | Kcnab2 | -0.62 |
| Slc22a12 | -1.14 | 1700027J07Rik | -0.82 | Tmem203 | -0.7 | Foxj2 | -0.62 |
| Nrxn1 | -1.1 | Slc12a7 | -0.82 | Ntsr2 | -0.69 | Prl3c1 | -0.62 |
| Gpr3 | -1.1 | 4933402D24Rik | -0.81 | Ttc9b | -0.69 | Cby3 | -0.62 |
| Atp2b2 | -1.09 | Cst7 | -0.81 | Rptor | -0.68 | Mlph | -0.62 |
| Ceacam10 | -1.08 | Npr3 | -0.81 | Hapln2 | -0.68 | Sh2b2 | -0.62 |
| Dscaml1 | -1.08 | Kalrn | -0.81 | Park2 | -0.68 | Dach1 | -0.61 |
| Elavl4 | -1.06 | Cdk5r1 | -0.8 | Pkp3 | -0.68 | Itm2a | -0.61 |
| Gm10308 | -1.05 | Chst2 | -0.8 | Zfp664 | -0.67 | 2610018G03Rik | -0.6 |
| Angptl2 | -1.04 | Mall | -0.79 | BC026439 | -0.67 | B4galnt2 | -0.6 |
| Slamf9 | -0.99 | Tnfrsf10b | -0.78 | Klk1b3 | -0.67 |  |  |
| Stap2 | -0.99 | Pcsk2 | -0.78 | Nnmt | -0.67 |  |  |
| Dact3 | -0.98 | Wnt2b | -0.78 | Rgnef | -0.67 |  |  |
| 4930447A16Rik | -0.98 | Jag1 | -0.77 | Figla | -0.66 |  |  |
| Def6 | -0.98 | Hs6st2 | -0.76 | Chrna7 | -0.66 |  |  |
| Bmp7 | -0.98 | Hipk2 | -0.76 | Exosc2 | -0.66 |  |  |
| Parvg | -0.96 | LOC100047123 | -0.75 | Itga2 | -0.66 |  |  |
