# Supplementary material for: TMEM203 Is a Novel Regulator of Intracellular Calcium Homeostasis and Is Required for Spermatogenesis
Source: PLoS One. 2015 May 21;10(5):e0127480. doi: 10.1371/journal.pone.0127480 (PMC4440627; doi:10.1371/journal.pone.0127480)
Supplement: S3 Table — Only genes with Avg Log2 FC >0.5 are shown. (DOCX) [file pone.0127480.s010.docx]

**Supplementary Table S3** – List of genes up-regulated in *Tmem203* null mouse testes

| Gene Symbol | Avg Log2 FC | Gene Symbol | Avg Log2 FC | Gene Symbol | Avg Log2 FC | Gene Symbol | Avg Log2 FC |
| --- | --- | --- | --- | --- | --- | --- | --- |
| Mpzl2 | 3.31 | Abca1 | 0.85 | Pcdh17 | 0.71 | Atp1b2 | 0.64 |
| Timp1 | 1.89 | Slc6a8 | 0.83 | Svs5 | 0.71 | Lgr4 | 0.64 |
| Robo4 | 1.84 | Nav1 | 0.83 | Pkia | 0.7 | Hsd3b1 | 0.64 |
| Ildr2 | 1.49 | Lcp1 | 0.82 | Smad3 | 0.7 | Ncoa7 | 0.64 |
| Fam155a | 1.31 | Ankrd43 | 0.82 | Cd38 | 0.7 | Ddr1 | 0.63 |
| Gfra1 | 1.27 | Apoc1 | 0.82 | Nrk | 0.7 | Igf1 | 0.63 |
| Fst | 1.22 | 1700025G04Rik | 0.81 | Ptprd | 0.69 | Sh3rf1 | 0.63 |
| Trim2 | 1.18 | Klhl14 | 0.81 | Jup | 0.69 | Tgm2 | 0.63 |
| Tnfrsf12a | 1.17 | Syt7 | 0.8 | Lipa | 0.69 | Pcyt1b | 0.63 |
| Klrb1a | 1.14 | Il1r1 | 0.8 | Smarca1 | 0.69 | Slc7a8 | 0.63 |
| Ttr | 1.14 | Sema5a | 0.8 | Usp9x | 0.69 | Dmd | 0.63 |
| Igfbp3 | 1.12 | Lgmn | 0.79 | Ltbp1 | 0.69 | Gpr56 | 0.63 |
| Zfp704 | 1.11 | Rbm47 | 0.79 | Nt5e | 0.69 | Htra1 | 0.63 |
| Flt4 | 1.1 | Pak3 | 0.78 | Sgms2 | 0.69 | Gcom1 | 0.62 |
| Ly6d | 1.09 | Cyp2c55 | 0.78 | Casp8 | 0.69 | Sox8 | 0.62 |
| Svs6 | 1.06 | Alcam | 0.78 | Mtap1b | 0.69 | Stard8 | 0.62 |
| 4930486L24Rik | 1.05 | Igf1r | 0.77 | Gm4926 | 0.68 | Lipg | 0.62 |
| Gjb2 | 1.04 | Lrrk1 | 0.77 | Kcnk6 | 0.68 | Ttc7b | 0.62 |
| Hspb8 | 1.04 | Ggta1 | 0.76 | Prlr | 0.68 | Diap2 | 0.62 |
| Fetub | 1.03 | Ppp1r3b | 0.76 | BC021891 | 0.68 | Vsig1 | 0.62 |
| Myoz2 | 1.02 | Hbb-y | 0.75 | Vwa5a | 0.67 | Ypel2 | 0.62 |
| Mkx | 0.99 | Pdgfra | 0.75 | Psg16 | 0.67 | Avpr1a | 0.62 |
| Spink8 | 0.97 | Nxnl2 | 0.75 | Rhoq | 0.67 | Eda2r | 0.62 |
| 5430407P10Rik | 0.97 | Timp2 | 0.75 | Daglb | 0.67 | Enc1 | 0.62 |
| Scn3b | 0.95 | Ldlr | 0.75 | Cxcr4 | 0.67 | Lrp4 | 0.62 |
| Camk2d | 0.94 | Gpr176 | 0.75 | Hpgd | 0.67 | Mllt6 | 0.62 |
| Gad2 | 0.93 | Tnrc6b | 0.75 | Ly6e | 0.66 | Aff1 | 0.61 |
| Ephb1 | 0.92 | Rab3b | 0.74 | AI987986 | 0.66 | Hivep2 | 0.61 |
| Pdcd1lg2 | 0.92 | Serpina3g | 0.74 | Zdhhc9 | 0.66 | Dpyd | 0.61 |
| Mmp9 | 0.9 | D3Bwg0562e | 0.74 | Sh3bp5 | 0.66 | Lpp | 0.61 |
| Crim1 | 0.89 | Arhgap31 | 0.74 | 9830001H06Rik | 0.65 | Ly96 | 0.61 |
| Il13ra1 | 0.89 | Chi3l3 | 0.73 | Gpt2 | 0.65 | Cgnl1 | 0.61 |
| Tspan8 | 0.88 | 4831426I19Rik | 0.73 | Ldhd | 0.65 | Klhl2 | 0.61 |
| Emb | 0.88 | Gpm6b | 0.73 | Shbg | 0.65 | Rnd3 | 0.61 |
| Tns3 | 0.88 | Stxbp6 | 0.72 | Tmprss13 | 0.65 | Esr1 | 0.61 |
| Cadps | 0.88 | Hsd17b11 | 0.72 | Lancl3 | 0.65 | Npl | 0.61 |
| Aplp1 | 0.87 | Arhgap26 | 0.72 | Itga6 | 0.65 | Peg10 | 0.61 |
| Pak6 | 0.86 | Ptprn2 | 0.72 | Kit | 0.65 | Lrch1 | 0.6 |
| Bhlhe40 | 0.85 | Klhl29 | 0.71 | Klf6 | 0.64 | Egr2 | 0.6 |
| Slc47a1 | 0.85 | Chga | 0.71 | Snord123 | 0.64 |  |  |
